# The Missing Piece of the Puzzle: Unveiling the Role of *PTPN11* Gene in Multiple Osteochondromas in a Large Cohort Study

**DOI:** 10.1155/2024/8849348

**Published:** 2024-02-12

**Authors:** Artem Borovikov, Nailya Galeeva, Andrey Marakhonov, Aysylu Murtazina, Varvara Kadnikova, Kseniya Davydenko, Anna Orlova, Peter Sparber, Tatiana Markova, Maria Orlova, Darya Osipova, Tatyana Nagornova, Natalia Semenova, Olga Levchenko, Alexandra Filatova, Margarita Sharova, Peter Vasiluev, Ilya Kanivets, Denis Pyankov, Artem Sharkov, Vasilisa Udalova, Vladimir Kenis, Natalia Nikitina, Maria Sumina, Konstantin Zherdev, Aleksandr Petel'guzov, Oleg Chelpachenko, Pavel Zubkov, Ivan Dan, Andrey Snetkov, Alexandra Akinshina, Yury Buklemishev, Oxana Ryzhkova, Vyacheslav Tabakov, Ekaterina Zakharova, Sergey Korostelev, Rena Zinchenko, Mikhail Skoblov, Alexander Polyakov, Elena Dadali, Sergey Kutsev, Olga Shchagina

**Affiliations:** ^1^Research Centre for Medical Genetics, Moscow, Russia; ^2^Genomed, Moscow, Russia; ^3^Russian Medical Academy of Continuous Professional Education, Moscow, Russia; ^4^Veltischev Research and Clinical Institute of Pediatrics and Pediatric Surgery of the Pirogov Russian National Research Medical University, Moscow, Russia; ^5^The Turner Scientific Research Institute for Children's Orthopedics, Saint Petersburg, Russia; ^6^State Healthcare Institution of Sverdlovsk Region “Clinical and Diagnostic Center “Mother's and Child Health Protection”, Ekaterinburg, Russia; ^7^National Medical Research Center of Children's Health, Moscow, Russia; ^8^I.M. Sechenov First Moscow State Medical University, Moscow, Russia; ^9^National Medical Research Center of Traumatology and Orthopedics Named after N.N. Priorov, Moscow, Russia

## Abstract

This study is aimed at investigating the clinical and genetic characteristics of 244 unrelated probands diagnosed with multiple osteochondromas (MO). The diagnosis of MO typically involves identifying multiple benign bone tumors known as osteochondromas (OCs) through imaging studies and physical examinations. However, cases with both OCs and enchondromas (ECs) may indicate the more rare condition metachondromatosis (MC), which is assumed to be distinct disease. Previous cohort studies of MO found heterozygous loss-of-function (LoF) variants only in the *EXT1* or *EXT2* genes, with DNA diagnostic yield ranging from 78 to 95%. The *PTPN11* gene, which is causative for MC, was not previously investigated as a gene candidate for MO. In this study, we detected a total of 177 unique single nucleotide and copy number variants in three genes across 220 probands, consisting of 80 previously reported and 97 novel variants. Specifically, we identified five cases with OCs and no ECs as well as four cases with MC carrying LoF variants in the *PTPN11* gene and two additional cases with ECs harboring variants in the *EXT1/2* genes. These findings suggest a potential overlap between the MO and MC both phenotypically and genetically. These findings highlight the importance of expanding genetic testing beyond the *EXT1* and *EXT2* genes in MO cases, as other genes such as *PTPN11* may also be causative. This can improve the accuracy of diagnosis and treatment for individuals with MO and MC. It is essential to determine whether MO and MC represent distinct diseases or if they encompass a broader clinical spectrum.

## 1. Introduction

Multiple osteochondromas (OMIM#133700, 133701) (MO) are the autosomal dominant skeletal dysplasia caused by heterozygous loss-of-function (LoF) variants in *EXT1* or *EXT2* genes with penetrance 98% [1, 2]. MO is characterized by the development of osteochondromas (OCs) which are benign bone tumors commonly located in the metaphyseal region of long bones and/or on the surface of flat bones. The identification of OCs through imaging studies such as X-rays or MRIs typically eliminates the need for differential diagnoses, and the presence of two or more OCs is sufficient for the diagnosis of MO according to clinical criteria [3]. The specificity and clear clinical findings of MO even make it possible to diagnose the disease by sequencing ancient DNA samples [4]. Despite the distinctive clinical features of MO, some families with MO still face challenges in obtaining a molecular diagnosis.

The *EXT1* and *EXT2* genes encode for glycosyltransferases exostosin-1 and exostosin-2, respectively, which are crucial for the synthesis and elongation of heparan sulfate (HS) [1, 2, 5], a sulfated glycosaminoglycan that plays an important role in the regulation of various cellular signaling pathways, including those involved in bone formation and growth [6]. The complete chain of molecular events linking the changes in HS synthesis by causative variants in the *EXT1/2* genes and the formation of OCs is still unknown. One hypothesis suggests that it could involve a second somatic hit leading to complete loss of one of the *EXT1/2* genes [7, 8]. However, studies of OC samples have revealed loss of heterozygosity in the *EXT1* gene in less than 10% of tumor samples, and this hypothesis cannot fully explain the high penetrance and greater number of OCs observed in patients compared to other monogenic diseases that rely on a second-hit mechanism [9, 10]. Several studies have suggested that deregulation of signaling pathways involved in normal bone formation can lead to the development of OCs through alterations in the level of HS [11, 12]. However, this hypothesis has some limitations as other genes, such as *EXTL1*, *EXTL2*, and *EXTL3*, which are also assumed to be involved in HS synthesis, and other genes involved in chondrogenesis have been investigated in many studies without any relevant findings [13–16]. Therefore, the role of HS and its related pathways in the formation of OCs remains an area of ongoing research and investigation.

Pathogenic variants in *EXT1/2* genes are found in a high percentage of individuals diagnosed with MO, with a range of 78-95%. DNA analysis of the coding region and copy number variations (CNVs) in *EXT1/2* genes is currently the gold standard for diagnosing MO [16–20]. The most commonly reported causative variants in the *EXT1/2* genes are nonsense, frameshift, and canonical splice site variants [21]. Functional studies of some of these variants have shown that the mutant transcripts undergo nonsense-mediated decay, leading to reduced expression of the EXT proteins [22, 23].

Despite extensive research, no other genes have been identified as causative for MO since *EXT1/2* genes were identified. Although another locus named *EXT3* was described, no single nucleotide variant (SNV) or CNV has been reported in patients with MO in 30 years after linkage analysis in that chromosomal region [24]. Noncoding variants in the *EXT1/2* genes could potentially explain undiagnosed cases of MO, but to date, all known noncoding pathogenic variants in these genes have been found in or near canonical splice sites [21]. Therefore, the identification and resolving of undiagnosed cases of MO may require genome sequencing or RNA analysis studies. Another potential explanation for missing heritability is the presence of somatic variants in the *EXT1/2* genes. It is worth noting that identifying somatic events can be particularly difficult due to the potential for low levels of mosaicism in patient blood samples. Therefore, accurate identification of somatic variants may require higher read coverage or analysis of different tissues. Several cases with mosaic CNV in the *EXT1/2* gene were published in association with the typical clinical presentation of MO, indicating the importance of exploring also somatic events in the context of MO [14, 20]. Additionally, there may be the possibility of another causative gene for MO that has yet to be identified.

Metachondromatosis (OMIM#156250) (MC) is a rare genetic disease characterized by the presence of obligatory enchondromas (ECs) and nonobligatory OCs, caused by LoF variants in the *PTPN11* gene [25, 26]. Conversely, gain-of-function pathogenic variants in *PTPN11* are associated with distinct diseases such as the Noonan syndrome (OMIM#163950) and multiple lentigines syndrome (OMIM#151100) [27, 28]. Studies of *Ptpn11* knock-out mice have demonstrated alterations in the Indian hedgehog (Ihh) pathway, which is also implicated in MO pathogenesis [29, 30]. MC is an extremely rare disease, with fewer than 60 cases reported worldwide, and only 17 cases have undergone molecular genetic testing in contrast to thousands of cases of MO [25, 26, 31]. Given the rarity of MC and its similarities to MO, the *PTPN11* gene represents a potential candidate gene for MO cases in which no pathogenic variants in the *EXT1/2* genes have been identified.

## 2. Materials and Methods

### 2.1. Patient Selection and Clinical Data

#### 2.1.1. Study Design

Our study encompasses two distinct patient groups based on the availability of clinical data. The first group consists of 226 probands who underwent comprehensive clinical evaluations at the Research Centre for Medical Genetics, providing sufficient clinical data for the diagnosis of MO or MC based on typical clinical and/or radiographic findings. CNV syndromes with MO as part of the phenotype were excluded [3]. Data from this group are employed for the analysis of clinical information and the calculation of diagnostic yield.

The second group comprises an additional 18 patients who lacked detailed clinical data but with novel findings in *EXT1/2* genes. Combining this group with the first, we have a total of 244 probands used for variant spectrum analysis. Among them, 201 individuals were of Russian descent, while 43 represented various ethnic minorities in Russia. Ethnic origins were determined based on self-reporting.

#### 2.1.2. Collected Clinical Data

We collected the following clinical information: age, sex, family history, age of discovery of the first osteochondroma, location of the first osteochondroma, number of skeletal sites with osteochondromas, assessment of upper and lower limb deformities, and assessment of range of motion at the major joints of the upper and lower limbs. We followed the familial history up to four generations.

We used the scale of severity by the Istituto Ortopedico Rizzoli (IOR) classification [32] and its revised version [33]. We performed assessments according to the old and new versions of the IOR scale separately. For gene-phenotype correlation, we used the revised version. We also use IOR scale for all *PTPN11*-related cases to address severity of disease and compare it with *EXT1/2*-related cases.

We had a nonuniform distribution of data availability in familial cases, as we had significant more clinical evaluation data from affected family members in some families than others. To exclude possible interference from other genetic factors for clinical correlation, we included only one youngest patient with sufficient clinical data from the family. A summary of the clinical and demographic data of the cohort is presented in Table 1.

### 2.2. DNA Analysis

**Figure 1:**
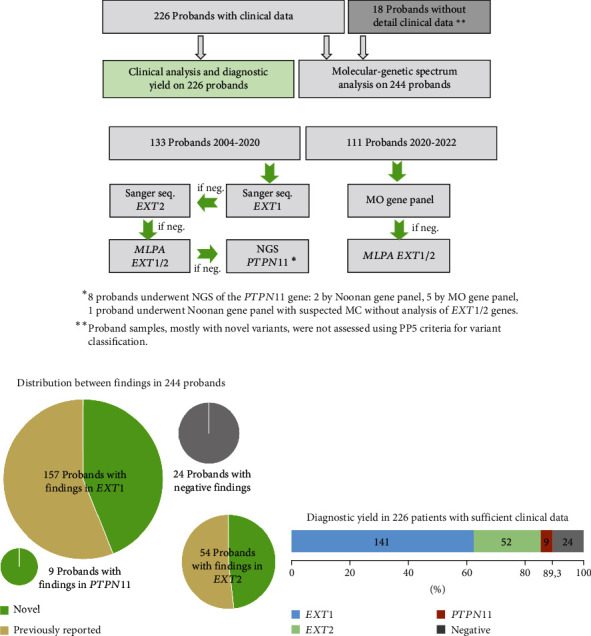
Design of the study and distribution between genetic findings. (a) Information about size of proband groups included in this study and information about usage of their data for different types of analysis. (b) Diagnostic pipeline. (c) Variants spectrum analysis between *EXT1/2*, *PTPN11* genes, and probands with negative findings. (d) Diagnostic yield of current study.

DNA was extracted from whole blood samples using a Wizard® Genomic DNA Purification Kit (Promega, USA). The Sanger sequencing was performed using ABI Prism 3500 Genetic Analyzer (Applied Biosystems, Foster City, CA, USA), with primer sequences designed based on the NM_000127.3 (*EXT1*), NM_207122.2 (*EXT2*), and NM_002834.5 (*PTPN11*) reference sequences. A next-generation sequencing (NGS) panel comprising 3 genes (*EXT1*, *EXT2*, and *PTPN11*) was used for sequencing on an Ion S5 (Thermo Fisher Scientific, Waltham, MA, USA) or MiSeq (Illumina, USA) sequencer. CNV analysis was carried out using the SALSA MLPA Probemix P215-B4 EXT (MRC Holland). A NGS panel for the Noonan syndrome comprising 18 genes (*MAP2K2*, *PPP1R13L*, *HRAS*, *NRAS*, *LZTR1*, *SHOC2*, *RASA2*, *CBL*, *NEK1*, *SOS1*, *SOS2*, *PTPN11*, *IFT80*, *A2ML1*, *BRAF*, *NF1*, *WDR35*, and *RASA1*) was performed for 3 patients. The findings from gene panels were confirmed by the Sanger sequencing. Covered regions by Sanger and gene panel sequencing are listed in Suppl. Table S1. The molecular diagnosis pipeline is illustrated in Figure 1(b). Information about performed tests for each proband is listed in Suppl. Table S2.

### 2.3. Bioinformatic Analysis

Bioinformatic analysis was performed using an in-house software pipeline as described earlier with modifications [34]. In brief, the pipeline involved quality control of raw reads using the FastQC tool v. 0.11.5, followed by read mapping to the hg19 human genome assembly using minimap2 v. 2.24-r1122. The alignments were sorted, and duplicates were marked using Picard Toolkit v. 2.18.14. Base recalibration and variant calling were performed with GATK3.8, and variant annotation was done using ANNOVAR tool (v.2018Apr16). The population frequencies of the identified variants were assessed in gnomAD v2.1.1. The variants were further filtered by functional consequences and population frequencies in accordance with the ACMG recommendations.

### 2.4. Search for LoF Variants in Local Exome/Genome Database

Search was performed in NGS dataset from probands with suspected genetic disease with inclusion healthy/affected relatives as part of trio analysis. The dataset includes whole exome and whole genome sequencing data, which consist of 51,214 alleles. We search LoF variants by applying filter by predicted effect: frameshift, nonsense, and variant near splice site in *EXT2* gene.

### 2.5. RNA Analysis

Analysis of the patient's mRNA structure was performed on primary cultured fibroblasts or mononuclear cell fraction extracted from the peripheral blood (PBMCs). Total RNA was extracted using the ExtractRNA reagent (Evrogen, Russia) according to the manufacturer's recommendations. Reverse transcription was performed using the ImProm-II™ Reverse Transcription System (Promega, USA).

For RNA analysis of the c.933+2dup variant in intron 8 of the *PTPN11*, primers specific to exons 5 and 10 of the *PTPN11* gene were used. The obtained PCR products were analyzed by gel electrophoresis followed by the Sanger sequencing. The deep next-generation sequencing of the PCR products was performed on an Ion Torrent S5 (with coverage > 200,000). The raw sequencing data was analyzed using an in-house bioinformatic pipeline based on open-source tools HISAT2, SAMtools, and SAJR. Splice junctions were visualized using Sashimi plot in IGV browser to identify any aberrant mRNA isoforms.

### 2.6. Visualization of Genetic Findings on Gene Scheme

Mapping of genetic findings on gene scheme was performed using MutationMapper from cBioPortal with additional further editing pictures [35].

### 2.7. Statistical Analysis

Statistical analysis was performed using GraphPad Prism 8.0.1 for Windows (GraphPad Software, San Diego, California, USA). *p* value less than 0.01 is considered to be statistically significant.

## 3. Results

### 3.1. Genetic Data Analysis

**Figure 2:**
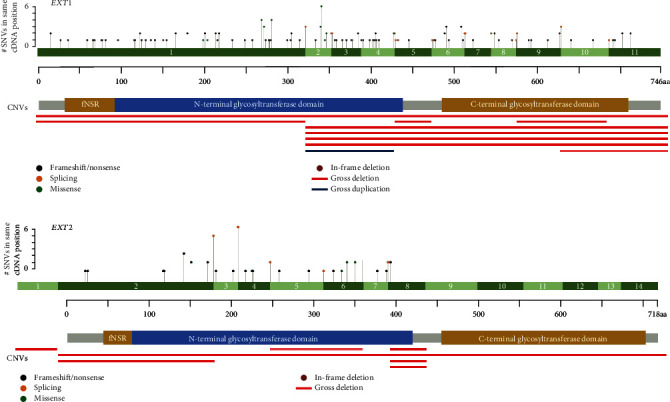
Detected variants in the *EXT1* and *EXT2* genes. Distribution of CNVs and CNVs on simplified scheme of *EXT1* and *EXT2* genes with relative accurate proportion between size of exon and protein domains. fNSR: flexible N-terminal stem region.

**Figure 3:**
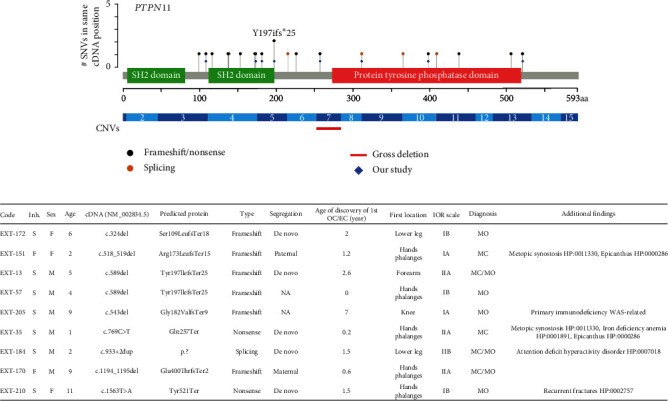
SNVs and CNVs in *PTPN11* gene associated with MC or MO. (a) Detected variants in our cohort and all record with MC from HGMD v.2022.1 database. SH2: Src homology 2. (b) Clinical summary of all probands with SNV in the *PTPN11* gene from our cohort. Inh.: inheritance; all cases that are not de novo: a variant inherited from a parent with signs of the disease; NA: not available for families that we could not receive parent blood sample for segregation analysis.

Clinical and genetic data were collected from 244 unrelated probands (146 males, 98 females) with initial diagnosis of MO. Of these, 121 cases were familial, 106 were sporadic, and 17 had no available family history. Following DNA diagnosis, causative variants in the *EXT1* gene were identified in 157 probands, while in 54 cases, causative variants were found in the *EXT2* gene (Figure 2). Additionally, LoF variants in the *PTPN11* gene were discovered in 9 patients (Figure 3(a)). Nineteen probands had no potential causative variant in *EXT1/2* and *PTPN11* genes, and five more underwent only the Sanger sequencing and MLPA of *EXT1/2* genes with negative results. In total, 177 unique SNVs or CNVs in *EXT1*, *EXT2*, and *PTPN11* genes were found. Among them, 97 are novel and 80 are previously reported. 32 variants appeared to be recurrent in our cohort, and 7 of them were novel. Most variants were classified according to ACMG criteria as pathogenic (PAT) or likely pathogenic (LPAT). Detailed molecular findings are present in Suppl. Table S2.

SNVs in the *EXT1* were found in 147 probands (Figure 2): 52 frameshift (35.4%), 42 nonsense (28.6%), 26 missense (17.7%), 24 variants in canonical splice sites (16.3%), and 3 in-frame deletion (2.0%). In the *EXT2* gene, causative SNVs were found in 48 cases: 8 frameshift (17.0%), 14 nonsense (29.8%), 7 missense (14.9%), and 18 splice cite variants (38.3%). Only 3 novel SNVs in our study are variants of uncertain significance (VUS): missense variant p.Leu335Ser in the *EXT2* gene and in-frame deletion p.Asp432_Ile434delinsGlu and missense variant p.Gly198Val in the *EXT1* gene. In all latter cases, segregation analyses or functional study could not be performed for their reclassification. In addition, 17 probands have CNVs in *EXT1* or *EXT2* genes: 16 gross deletions and 1 gross duplication. Of these, 3 gross deletions of exon 8 in the *EXT2* gene in 3 families (1 sporadic and 2 familial) and 4 deletions of exons 2–11 of the *EXT1* gene in 4 families (3 sporadic and 1 familial) were considered as possibly recurrent since the exact breakpoints cannot be determined by MLPA. Two novel CNVs were identified in the *EXT1* gene and were classified as VUS in our study. One CNV is a deletion of exons 5-6 in-frame (#EXT-83), while the other is a deletion of the last exons 10-11 (#EXT_nd-28). While these CNVs could potentially lead to a partial gene function, the likelihood of this outcome is considered to be low, but they still cannot be reclassified without segregation study or functional analysis.

**Figure 4:**
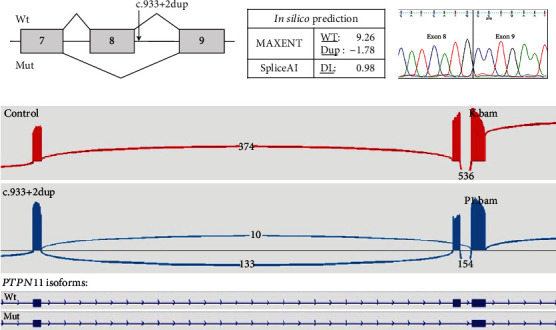
Functional study for the case #EXT-184. (a) Scheme of exon skipping due to c.933+2dup variant in the *PTPN11* gene. (b) Prediction of the effect on splicing mRNA of the *PTPN11* gene caused by c.933+2dup variant *in silico*. (c) The Sanger sequencing of PCR product. (d) Target deep NGS and isolation for IGV browser.

Even though all patients were referred with initial diagnosis of MO, 3 of them (##EXT-13, EXT-35, and EXT-151) had additional nontypical for MO clinical picture: OC-like lesions in hands and less severe course of disease. Following the negative results of *EXT1/2* gene analysis in two patients (#EXT-13, EXT-151), we re-evaluated their clinical presentation and found indications that they may have MC instead of MO. The proband #EXT-35 was reclassified from MO to MC before DNA diagnosis. These three patients underwent sequencing of the *PTPN11* gene by gene panel for the Noonan syndrome, which led to the discovery of LoF variants in each case. These findings prompted us to include the *PTPN11* gene in a gene panel for MO. The use of this panel led to identifying five additional patients with LoF variants in the *PTPN11* gene who had no obvious initial clinical signs of MC and ECs and one typical cases of MC with ECs. In total, two cases with *PTPN11* variants were familial while seven others were sporadic (including two probands with recurrent frameshift variant p.Tyr197IlefsTer25). All identified variants (6 frameshift, 2 nonsense, and 1 splicing) were novel and classified as pathogenic according to ACMG criteria (PVS1, PM2, and PP4). Segregation analysis, where available, provided evidence supporting the causative role of LoF variants in the *PTPN11* gene in the pathogenesis of MO (Figure 3(b)). In addition, functional analysis of the c.933+2dup variant was performed by RT-PCR analysis of the mRNA obtained from proband #EXT-184's cultured fibroblasts. Bioinformatic analysis showed that this variant with a high probability leads to the disruption of the donor splice site in intron 8 (Figure 4(b)) which is frequently accompanied by exon skipping. The Sanger sequencing of the obtained PCR product did not reveal any splicing abnormalities between analyzed exons of the *PTPN11* gene (Figure 4(c)). However, based on the in silico predictions, we assumed that the resulting aberrant transcript may contain a premature stop codon (PTC) and be almost completely degraded by NMD. Thus, the Sanger sequencing may not be sensitive enough to detect such transcript. To overcome this limitation, we additionally analyzed PCR product using target deep NGS. In the proband sample, we detected ~7% reads corresponding to the isoform with exon 8 skipping which were absent in the control sample (Figure 4(d)). Thus, the c.933+2dup variant disrupts the donor splicing site of *PTPN11* intron 8, resulting in exon 8 skipping and a frameshift. The frameshift produces a premature stop codon and leads to the mRNA degradation by NMD.

### 3.2. Clinical Data Analysis

**Figure 5:**
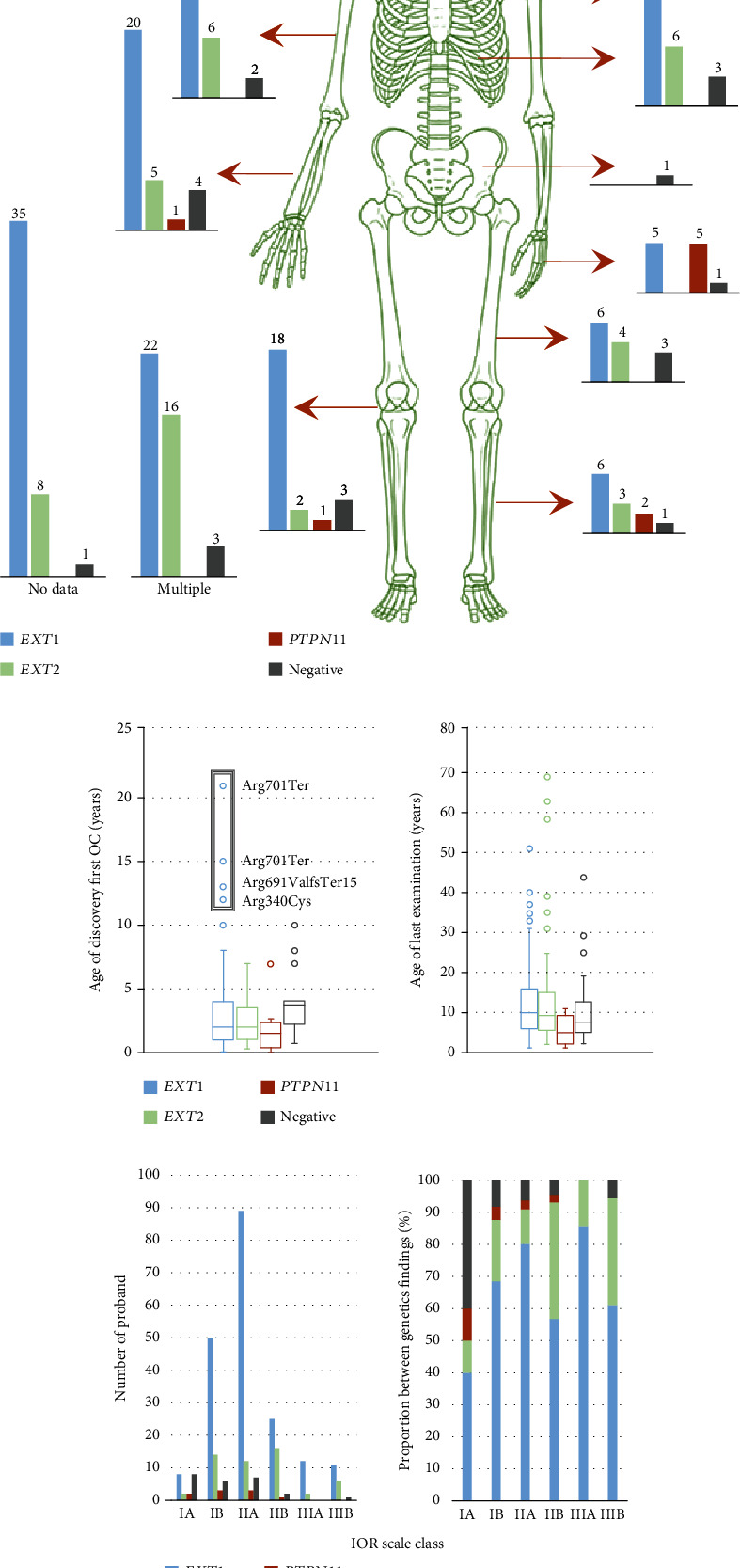
Clinical data of cohort. (a) Localization of the first discovered OC according to molecular findings. (b) Age of discovery of the first OC and age of last examination. All groups present according to result of DNA diagnostic with the same color choice as in (a). (c) Number and proportion of probands with different score of IOR severity scale, grouped according to genetic findings with the same color choice as in (a) and (b).

For gene-phenotype correlation analysis, we include only 226 patients of the youngest age (median 10 years, IQR 5.25-16) from unrelated families who underwent the standard clinical examination according to the IOR scale, detailed clinical data present in Suppl. Table S2. All 226 probands were divided into 3 major groups with 2 subgroups each and exclude other relatives to avoid nonuniformity of cohort (Figure 5(c)). The use of the old version of IOR scale resulted in underrepresentation of group IIB (5 probands) and overrepresentation of subgroup IIA (95 probands). When using the revised version, 48 probands were reclassified from IIA to IIB and resulted in more harmonious data for further analysis. Analysis between severity and molecular findings showed that distribution of probands between IOR groups as well as by age in cases with causative variants in *EXT1*, *EXT2*, and *PTPN11* genes and negative cases was not significantly different (*p* value = 0.4732) (Figure 5(c)). We did observe as expected that the median age of probands in severer groups is greater (*p* value < 0.0001).

We conducted an analysis of disease onset in our cohort by comparing the age of discovery of the first OC between different groups of probands. The analysis was performed for 193 probands for whom data were available (Figure 5(b)). Interestingly, the age of discovery of the first OC was before five years old in 90% of our probands, with a mean of 2.7 years and a median of 2.0 years, and there was no significant difference between genders (*p* value = 0.3228). We found that only 4 probands (1.6%) had their first OC discovered after the age of 12 years, with three of these patients having single SNVs in the last exon of the *EXT1* gene (Figure 5(b)). Two probands with the recurrent variant p.Arg701Ter had the latest onset at 15 and 21 years old, and one proband with p.Arg691ValfsTer15 had an onset at 13 years old. Interestingly, the patient #EXT-77 with the p.Arg701Ter variant in the last exon of the *EXT1* gene was the only case with malignization. The discovery of chondrosarcoma of the rib in this patient led to the identification of other small OCs in the limbs and established an MO diagnosis at the age of 15 years. The other two patients with variants in last 2 exons of the *EXT1* gene, p.Trp711Ter and p.Trp692Ter, had a typical age of discovery of the first OC, which was at 1.5 years of age. Of the remaining proband, #EXT-98 with late discovery of OCs in 12 age old had missense variant p.Arg340Cys in the *EXT1* gene in exon 2.

We conducted an analysis of the localization of the first discovered OC in our cohort of 200 cases where data were available (Figure 5(a)). Of these, 41 cases (20.6%) had multiple OCs found simultaneously. The majority of first discovered OCs were located in the ribs (16.6%), followed by the forearm (15.1%), the region of the knee (12.1%), arms (10.1%), upper (6.5%) and lower (6.0%) legs, scapula (6.5%), and hand (5.5%). Only one had the first discovered OC located in the clavicle (0.5%) and one case in the pelvis (0.5%). We compared the distributions of the first discovered OC in groups with different causative genes and negative cases, but no significant difference was observed (*p* value = 0.0469).

Among the 193 probands with confirmed genetic variants in the *EXT1/2* genes with sufficient clinical data, only two (#EXT-48, #EXT-183) were found to have ECs with OCs and carry a frameshift variant p.(Ser141ProfsTer16) and a deletion of exon 5 in the *EXT1* gene. All our probands with LoF variants in the *PTPN11* were additionally examined after the result of molecular diagnosis. Four out of 9 patients have typical for MO clinical findings, 3 more have some minor signs of MC (OC-like lesions with localization predominantly in hand), and 2 others have enchondromas and location of all lesions only in the hand and feet (Figure 3(b)).

### 3.3. Variant Distribution in the *EXT2* Gene

Upon mapping the variant distribution in the *EXT2* gene, we observed that all MO-associated variants in our cohort were confined to the first half of the gene, up to exon 9 out of a total of 14 (Figure 2(b)). To further investigate this disproportion, we searched for LoF variants (nonsense, frameshift, and canonical splice site variants) in the *EXT2* gene in our local database of exome and genome sequencing data, which included 51,214 alleles. We found 8 persons with LoF variants in the *EXT2* gene who did not have any reported OCs in their clinical records or family history of MO and had undergone genetic testing for another diagnosis (Suppl. Table S2). Two had variants located in exon 2, one of which (p.Leu71ArgfsTer41) was found in a fetal sample with multiple congenital abnormalities and the other known pathogenic variant (p.Arg23Ter) was identified in a 4-year-old girl with oculocutaneous albinism. These patients may be subclinical and could develop MO later on. The remaining 6 LoF variants were located after exon 8, and 5 of the 6 individuals were over 17 years old, and one was 3 years old. Based on the median age of discovery of the first OC, these 5 out of 6 individuals would have already manifested clinical symptoms if these variants were causative for MO. Two of the 8 patients were available and agreed to undergo clinical evaluation, but we did not identify any OCs or skeletal abnormalities in them.

## 4. Discussion

We conducted a comprehensive analysis of a large cohort of individuals with an initial diagnosis of MO and were able to establish the molecular cause in 89.3% of cases with sufficient clinical data (202 out 226) (Figure 1). Notably, probands who yielded negative results in our study did not exhibit significant differences in severity scores when compared to those with molecular confirmation. Our study, however, had certain limitations. Firstly, we restricted our analysis to the coding regions and closes noncoding to exon borders of the *EXT1/2* and *PTPN11* genes. Additionally, we did not include CNV analysis for the *PTPN11* gene. These constraints underscore the need for further investigations to explore noncoding variants, somatic mutations, or variants in other genes that may account for the condition in individuals with negative results. Additionally, we included 18 SNVs to expand our list of genetic findings. These 18 variants were identified in patients with limited clinical data, as some individuals only had information indicating a diagnosis of MO without further details (Figure 1). Our cohort of MO probands is consistent with other major studies with a comparable number of patients in more than 70 families, with no significant differences observed in the ratio of pathogenic variants in either *EXT1* or *EXT2* genes [13, 15–17, 36, 37].

Our findings expand the number of causative variants by 67 novel variants in the *EXT1* gene, 22 in the *EXT2* gene, and 8 in the *PTPN11* gene (Suppl. Table S2). Most novel variants were classified as PAT or LPAT according to ACMG criteria. A part of novel variants were classified as VUS based on the first results of DNA diagnosis and were subsequently reclassified as LPAT after segregation studies.

However, three novel SNVs and two CNVs could only be classified as VUS due to the unavailability of family members for further investigation. It is worth noting that all novel VUS SNVs and one CNV are located within functional domains, as determined by recent structural works [38, 39]. We also found the novel deletion in the *EXT1* gene, causing a stop-loss consequence by affecting the last 10-11 exons, in proband without any additional non-MO-related symptoms. Only one de novo deletion of last exon 11 of the *EXT1* gene was published before in Bulgarian MO-cohort, other deletions that include last exons had bigger size, and the smallest of them affects exons 4-11 [40]. In other work, the exact breakpoint of Bulgarian deletion exon 11 was identified, but without any functional studies about molecular consequence [41]. Although the identified deletion of the last exons, 10-11, is likely to result in a nonfunctional transcript, we do not know the exact breakpoints and segregation status. Therefore, at present, we can only classify this deletion as a VUS. However, it is intriguing to consider the small yet exciting possibility that this deletion could potentially lead to the production of a protein with a noncanonical stop codon.

Our analysis revealed 34 recurrent variants in 76 probands (31.1%), of which 51 cases were familial, 22 were sporadic, and 3 had no family history information. This distribution deviates from the nonrecurrent variant's distribution of familial and sporadic cases (51 : 22 vs. 69 : 61), suggesting distant relatedness of at least some probands with similar variants. Among these recurrent variants, the missense variant p.Arg340Leu in the *EXT1* gene was found in 5 families (4 with family history and 1 sporadic) and 6 different SNVs were found in three unrelated families each. Considering the recurrence of certain variants across multiple families, it would be valuable to investigate the possibility of shared ancestry among these families. Such analysis would shed light on whether the observed variants are a result of a common ancestors or not.

In our study, 9 out 226 (3.9%) of cases referred initially with MO were associated with LoF variants in the *PTPN11* gene (Figure 3). While the majority of reported patients with pathogenic variants in *PTPN11* gene are associated with Noonan and multiple lentigines syndromes, which have gain-of-function as a mechanism of molecular pathogenesis [27, 28], only 17 cases were linked MC to LoF variants in the *PTPN11* gene [25, 26]. Although it shares similarities with MO, MC is distinct in clinical picture in terms of lesion distribution, presence of ECs, and other imaging findings. Several MO patients have also been reported to exhibit features of MC, such as enchondromas in the hands; these observations have mostly been made without molecular confirmation except one case with frameshift variant in the *EXT2* gene [31, 42, 43]. In our cohort, 2 probands (#EXT-48, #EXT-183) with p.Ser141ProfsTer16 and gross deletion of exon 5 in the *EXT1* gene also have enchondromas of the hands in combination with OCs. Three probands in our studies did not have ECs but had OC-like lesions with small number of OCs in other bones. We position these cases within the intermediate range of the MC to MO phenotype spectrum. A similar clinical finding was previously described in a case of nine-year-old boy with LoF variant in the *PTPN11* gene who presented with OC-like lesions in the hands and feet with OCs in the tibiae, all without any enchondromas [44]. The overlap between the two diseases has been discussed in other studies [31, 43], and the current understanding of the molecular etiology of MC is limited, as only three studies have been published on this topic [25, 26, 44]. We also need to consider the young age of our probands with variants in the *PTPN11* gene and without ECs. There is a possibility that these patients might develop ECs in the future. Furthermore, the *PTPN11* gene has not been investigated in MO cohorts, which makes it difficult to determine whether MC and MO represent distinct disorders or different manifestations of a single disease with a broad clinical spectrum. Obtaining molecular confirmation for all MO and MC cases is essential for conducting future observational studies and drawing comprehensive conclusions about both diseases.

Therefore, our identification of LoF variants in the *PTPN11* gene, without the presence of other variants in *EXT1/2* genes, suggests that these variants may be causative for MO with or without MC features in our patients. However, the rarity of the presence of MC features in most MO patients with variants in the *EXT1/2* genes warrants further investigation. Moreover, the reasons why some patients with MO have MC features and why some patients with LoF variants in the *PTPN11* gene have or do not have MC features are not fully understood. The pathogenesis of MO in cases involving *PTPN11* and *EXT1/2* genes may be linked by their involvement in the Ihh pathway [29, 45]. Previous research has demonstrated that the Ihh pathway is altered in MO samples with variants in the *EXT1/2* genes possibly due to decreased synthesis of HS and was regarded as a potential therapeutic target for MO treatment [8, 45, 46]. Therefore, deletion of the *PTPN11* gene also activates the same pathway in the mouse model of MC [29].

It is worth mentioning that the most serious complication in MO is malignant transformation to a chondrosarcoma, occurring in 1%–5% of cases according to different studies [13, 15–17, 36, 37, 47]. We have only 1 confirmed case of malignant transformation with nonsense variant in the last exon of the *EXT1* gene (#EXT-77). And we also have reported case of malignant transformation in one family history (#EXT-190). High frequency of malignant transformation up to 20% of patients in other studies is mostly reported in cohorts from surgery departments [48]. Also, most of our probands have a juvenile age of examination, and it was noted that malignant transformation of OCs occurs more frequently in adult age [48].

The distribution pattern of LoF variants in the *EXT1/2* genes is an interesting finding that warrants further discussion. In patients with MO, LoF variants in the *EXT1* gene have been shown to occur across all exons, and these variants are significantly underrepresented in the gnomAD database (pLI score = 1) [49]. In contrast, in the probands of our cohort with MO, LoF variants in the *EXT2* gene were only located in the first half of the gene (before exon 9 out of 14). Despite haploinsufficiency being a primary pathogenic mechanism for MO, gnomAD data for *EXT2* has a pLI score of 0. The distribution of LoF variants in the gnomAD database demonstrates a strong depletion of variants in exons 1-8 (1306 b.p. in length) of the gene, compared to those located in exons 9-14 (947 b.p. in length) (21 vs. 42 alleles, *p* value = 5.9 × 10^−5^) [49]. We were unable to link the observed distribution to the “rules” of NMD escape or the presence of the known shorter isoform of *EXT2*. Other studies have consistently demonstrated that *EXT1* and *EXT2* proteins form a single enzyme, and disease-associated LoF variants in the *EXT2* gene lead to a reduction in the level of *EXT2* protein and NMD [7, 10, 22, 23, 38, 39, 50].

We conducted an analysis of a local database of exome and genome sequencing data (comprising 51,214 alleles) and identified eight cases with LoF variants in the *EXT2* gene who were referred for diagnoses other than MO and had no known family history of MO (Suppl. Table S3). These findings are well combined with population data and published variants in the *EXT2* gene. The HGMD v.2022.1 database contains records of only nine SNVs after exon 8 in the *EXT2* gene with MO, representing only a small fraction of the total 283 known variants in the *EXT2* gene [21]. Of these nine variants, five are missense and four are nonsense variants. Seven were published without any clinical data and the one case with two missense variants after ex8 with another frameshift variant in the *EXT1* gene. It is also worth noting that three variants described as disease-causative were present in the gnomAD v2.1 database with allele frequencies higher than expected for MO (on 9, 34, and 187 alleles) [49]. Based on the available evidence, it appears that variants occurring after exon 8 in the *EXT2* gene could be not causative for MO, for reasons that are currently unknown. Interestingly, there is another phenotype associated with the *EXT2* gene known as seizure, scoliosis, and macrocephaly/microcephaly (SSM) syndrome, which is caused by biallelic variants in the *EXT2* gene [51]. Some patients with SSM have also been observed to have OCs, but only when one of the variants is located in the first half of the gene [51]. However, the existence of SSM syndrome with a different inheritance pattern and a clinical picture distinct from MO does not shed much light on the mystery of variant distribution in the *EXT2* gene. In conclusion, the enigma of the *EXT2* gene does not seem to fit well with a simple mechanism of haploinsufficiency for autosomal dominant diseases and requires further investigation.

## 5. Conclusions

In conclusion, our research significantly expands our understanding of the genetic basis of MO by identifying 97 novel variants, increasing the allelic and locus heterogeneity of the disease. Furthermore, our study highlights the potential importance of investigating the *PTPN11* gene in undiagnosed MO cases, as it increased DNA diagnostic yield. We are particularly intrigued by the possibility of additional cases of MO being linked to LoF variants in the *PTPN11* gene in other patient cohorts from different countries, which could shed light on whether MO and MC are distinct diseases or one broader clinical spectrum. The observed pattern of variant distribution in the *EXT2* gene, as well as the relationship between the *PTPN11* gene and the *EXT1/2* genes, necessitates further investigation. Exploring these connections has the potential to provide valuable insights into the molecular mechanisms underlying the pathogenesis and clinical management of MO, offering promising avenues for future research.

## Figures and Tables

**Table 1:** Summary of clinical data from 244 probands from unrelated families.

	Male	Female	Total
Number	146	98	244
Sporadic cases	64	42	106
Familial cases	70	51	121
Unknown family history	12	5	17
Age of last examination (median, IQR)	9 (5-15)	10.5 (6-25)	10 (5.25-16)
Availability of clinical data for IOR scale	134	92	226
Not sufficient clinical data for IOR scale	12	6	18
Localization of first OC	122	78	200
Unknown localization	24	20	44
Age of discovery of 1st OC	118	75	193
Unknown age of discover of 1st OC	28	23	51

IQR: interquartile range; IOR: clinical scale of severity of MO by the Istituto Ortopedico Rizzoli.

## Data Availability

The datasets used and/or analyzed during the current study are available from the corresponding author on reasonable request.

## Supplementary Materials

Supplementary 1 Table S1: chromosomal positions of covered regions by the Sanger and gene panel sequencing.

Supplementary 2 Table S2: clinical and molecular data of 244 probands from unrelated families.

Supplementary 3 Table S3: clinical and molecular data from persons without any sights of osteochondromas from our local database with loss-of-function variant in the *EXT2* gene.
