# Supplementary material for: The Missing Piece of the Puzzle: Unveiling the Role of PTPN11 Gene in Multiple Osteochondromas in a Large Cohort Study
Source: Hum Mutat. 2024 Feb 12;2024:8849348. doi: 10.1155/2024/8849348 (PMC11918999; doi:10.1155/2024/8849348)
Supplement: Supplementary 1 — Table S1: chromosomal positions of covered regions by the Sanger and gene panel sequencing. [file 8849348.f1.docx]

**Suppl. Table S1. Part A. Chromosomal positions of covered regions by gene panels**

| Gene panel name | GRCh37_CHR | GRCh37_start | GRCh37_end | Gene_Symbol | HGVS_transcript | Exon # |
| --- | --- | --- | --- | --- | --- | --- |
| Gene panel MO | chr11 | 44117792 | 44117881 | EXT2 | NM_207122.2:c.-31+390_-31+479 | ex1 |
| Gene panel MO | chr11 | 44129222 | 44129808 | EXT2 | NM_207122.2:c.-30-9_536+12 | ex2 |
| Gene panel MO | chr11 | 44130733 | 44130843 | EXT2 | NM_207122.2:c.537-10_626+11 | ex3 |
| Gene panel MO | chr11 | 44135724 | 44135861 | EXT2 | NM_207122.2:c.627-9_743+12 | ex4 |
| Gene panel MO | chr11 | 44146328 | 44146544 | EXT2 | NM_207122.2:c.744-8_939+13 | ex5 |
| Gene panel MO | chr11 | 44148355 | 44148515 | EXT2 | NM_207122.2:c.940-11_1079+10 | ex6 |
| Gene panel MO | chr11 | 44151584 | 44151698 | EXT2 | NM_207122.2:c.1080-11_1173+10 | ex7 |
| Gene panel MO | chr11 | 44193150 | 44193302 | EXT2 | NM_207122.2:c.1174-10_1305+11 | ex8 |
| Gene panel MO | chr11 | 44219368 | 44219578 | EXT2 | NM_207122.2:c.1306-10_1495+11 | ex9 |
| Gene panel MO | chr11 | 44228332 | 44228519 | EXT2 | NM_207122.2:c.1496-10_1662+11 | ex10 |
| Gene panel MO | chr11 | 44253892 | 44254056 | EXT2 | NM_207122.2:c.1663-11_1806+10 | ex11 |
| Gene panel MO | chr11 | 44255654 | 44255803 | EXT2 | NM_207122.2:c.1807-11_1935+10 | ex12 |
| Gene panel MO | chr11 | 44257832 | 44257935 | EXT2 | NM_207122.2:c.1936-11_2018+10 | ex13 |
| Gene panel MO | chr11 | 44265688 | 44265847 | EXT2 | NM_207122.2:c.2019-11_*10 | ex14 |
| Gene panel MO | chr12 | 112856905 | 112856939 | PTPN11 | NM_002834.5:c.-11_14+10 | ex1 |
| Gene panel MO | chr12 | 112884069 | 112884212 | PTPN11 | NM_002834.5:c.15-11_137+10 | ex2 |
| Gene panel MO | chr12 | 112888111 | 112888326 | PTPN11 | NM_002834.5:c.138-11_332+10 | ex3 |
| Gene panel MO | chr12 | 112890988 | 112891201 | PTPN11 | NM_002834.5:c.333-10_525+11 | ex4 |
| Gene panel MO | chr12 | 112892357 | 112892494 | PTPN11 | NM_002834.5:c.526-10_642+11 | ex5 |
| Gene panel MO | chr12 | 112893743 | 112893877 | PTPN11 | NM_002834.5:c.643-11_756+10 | ex6 |
| Gene panel MO | chr12 | 112910737 | 112910854 | PTPN11 | NM_002834.5:c.757-11_853+10 | ex7 |
| Gene panel MO | chr12 | 112915444 | 112915544 | PTPN11 | NM_002834.5:c.854-11_933+10 | ex8 |
| Gene panel MO | chr12 | 112915650 | 112915829 | PTPN11 | NM_002834.5:c.934-11_1092+10 | ex9 |
| Gene panel MO | chr12 | 112919867 | 112920019 | PTPN11 | NM_002834.5:c.1093-11_1224+10 | ex10 |
| Gene panel MO | chr12 | 112924268 | 112924443 | PTPN11 | NM_002834.5:c.1225-9_1379+12 | ex11 |
| Gene panel MO | chr12 | 112926236 | 112926324 | PTPN11 | NM_002834.5:c.1380-10_1447+11 | ex12 |
| Gene panel MO | chr12 | 112926817 | 112926989 | PTPN11 | NM_002834.5:c.1448-11_1599+10 | ex13 |
| Gene panel MO | chr12 | 112939937 | 112940070 | PTPN11 | NM_002834.5:c.1600-10_1712+11 | ex14 |
| Gene panel MO | chr12 | 112942488 | 112942610 | PTPN11 | NM_002834.5:c.1713-11_*32+10 | ex15 |
| Gene panel MO | chr8 | 119122313 | 119123295 | EXT1 | NM_000127.3:c.-7_962+14 | ex1 |
| Gene panel MO | chr8 | 118849336 | 118849450 | EXT1 | NM_000127.3:c.963-10_1056+11 | ex2 |
| Gene panel MO | chr8 | 118847672 | 118847800 | EXT1 | NM_000127.3:c.1057-10_1164+11 | ex3 |
| Gene panel MO | chr8 | 118842458 | 118842598 | EXT1 | NM_000127.3:c.1165-10_1284+11 | ex4 |
| Gene panel MO | chr8 | 118834693 | 118834846 | EXT1 | NM_000127.3:c.1285-9_1417+12 | ex5 |
| Gene panel MO | chr8 | 118831904 | 118832043 | EXT1 | NM_000127.3:c.1418-10_1536+11 | ex6 |
| Gene panel MO | chr8 | 118830663 | 118830779 | EXT1 | NM_000127.3:c.1537-10_1632+11 | ex7 |
| Gene panel MO | chr8 | 118825100 | 118825210 | EXT1 | NM_000127.3:c.1633-9_1722+12 | ex8 |
| Gene panel MO | chr8 | 118819445 | 118819626 | EXT1 | NM_000127.3:c.1723-10_1883+11 | ex9 |
| Gene panel MO | chr8 | 118816950 | 118817142 | EXT1 | NM_000127.3:c.1884-9_2055+12 | ex10 |
| Gene panel MO | chr8 | 118811940 | 118812146 | EXT1 | NM_000127.3:c.2056-10_*11 | ex11 |
| Gene panel Noonan | chr12 | 112856905 | 112856939 | PTPN11 | NM_002834.5:c.-11_14+10del | ex1 |
| Gene panel Noonan | chr12 | 112884069 | 112884212 | PTPN11 | NM_002834.5:c.15-11_137+10del | ex2 |
| Gene panel Noonan | chr12 | 112888111 | 112888326 | PTPN11 | NM_002834.5:c.138-11_332+10del | ex3 |
| Gene panel Noonan | chr12 | 112890988 | 112891201 | PTPN11 | NM_002834.5:c.333-10_525+11del | ex4 |
| Gene panel Noonan | chr12 | 112892357 | 112892494 | PTPN11 | NM_002834.5:c.526-10_642+11del | ex5 |
| Gene panel Noonan | chr12 | 112893743 | 112893877 | PTPN11 | NM_002834.5:c.643-11_756+10del | ex6 |
| Gene panel Noonan | chr12 | 112910737 | 112910854 | PTPN11 | NM_002834.5:c.757-11_853+10del | ex7 |
| Gene panel Noonan | chr12 | 112915444 | 112915544 | PTPN11 | NM_002834.5:c.854-11_933+10del | ex8 |
| Gene panel Noonan | chr12 | 112915650 | 112915829 | PTPN11 | NM_002834.5:c.934-11_1092+10del | ex9 |
| Gene panel Noonan | chr12 | 112919867 | 112920019 | PTPN11 | NM_002834.5:c.1093-11_1224+10del | ex10 |
| Gene panel Noonan | chr12 | 112924268 | 112924443 | PTPN11 | NM_002834.5:c.1225-9_1379+12del | ex11 |
| Gene panel Noonan | chr12 | 112926236 | 112926324 | PTPN11 | NM_002834.5:c.1380-10_1447+11del | ex12 |
| Gene panel Noonan | chr12 | 112926817 | 112926989 | PTPN11 | NM_002834.5:c.1448-11_1599+10del | ex13 |
| Gene panel Noonan | chr12 | 112939937 | 112940070 | PTPN11 | NM_002834.5:c.1600-10_1712+11del | ex14 |
| Gene panel Noonan | chr12 | 112942488 | 112942610 | PTPN11 | NM_002834.5:c.1713-11_*32+10del | ex15 |

**Suppl. Table S1. Part B. Chromosomal positions of covered regions by Sanger sequencing**

| Forward primer name | Forward primer sequence | Reverse primer name | Reverse primer sequence | GRCh37_CHR | GRCh37_start | GRCh37_end | Gene_Symbol | Covered HGVS_transcript | Exon # |
| --- | --- | --- | --- | --- | --- | --- | --- | --- | --- |
| EXT1 F 1-1 | TTGATTGGGAAACTTGGGTGATTC | EXT1 R 1-1 | TCTCTGTCTAAAGTATCCAGACTC | chr8 | 119122795 | 119123348 | *EXT1* | NM_000127.3:c.-63_491 | ex1 |
| EXT1 F 1-2 | CGAGGGCTCCAGGTTCTACAC | EXT1 R 1-2 | CGGCAGAGCCCAAGGCTGAC | chr8 | 119122274 | 119122872 | *EXT1* | NM_000127.3:c.414_962+50 | ex1 |
| EXT1 F2 | GTTGCTTTGCGTAAATTCATGCAC | EXT1 R2 | GTTAAACCCACTTAATCTGGCTTC | chr8 | 118849282 | 118849542 | *EXT1* | NM_000127.3:c.963-102_1056+65 | ex2 |
| EXT1 F3 | ACAGCTTCTGCTGTCGCTTTCC | EXT1 R3 | GATTCATCTTCTTTGAAAGTTTGGAC | chr8 | 118847606 | 118847843 | *EXT1* | NM_000127.3:c.1057-53_1164+77 | ex3 |
| EXT1 F4 | GGGTTATTTTGATCAAGTGCATCTC | EXT1 R4 | GCTGAGAGAAGTGTATAAAGGACC | chr8 | 118842409 | 118842658 | *EXT1* | NM_000127.3:c.1165-70_1284+60 | ex4 |
| EXT1 F5 | CCAAATATCATCAGGATCTCATCC | EXT1 R5 | GCCTTTAGTTCTGTATGACATCTTC | chr8 | 118834639 | 118834888 | *EXT1* | NM_000127.3:c.1285-51_1417+66 | ex5 |
| EXT1 F6 | TAAGTCTTGCTTTCCAGCGCTTC | EXT1 R6 | CTGGAGCAGGCAGGGGCTTC | chr8 | 118831875 | 118832085 | *EXT1* | NM_000127.3:c.1418-52_1536+40 | ex6 |
| EXT1 F7 | AGGCTTTGGGTTGGAGGCATAC | EXT1 R7 | CCAAGGCTCCACAGTGGTTCC | chr8 | 118830627 | 118830838 | *EXT1* | NM_000127.3:c.1537-69_1632+47 | ex7 |
| EXT1 F8 | AAGACTCTGAAGTTACCTCTTTCC | EXT1 R8 | CAACATGAGGTGACTGCCTGAAC | chr8 | 118825053 | 118825263 | *EXT1* | NM_000127.3:c.1633-63_1722+58 | ex8 |
| EXT1 F9 | GGGAGAAGGTAATGTTTTGTTGAC | EXT1 R9 | GCCTTAGTTCCTATTTATGCAGC | chr8 | 118819379 | 118819688 | *EXT1* | NM_000127.3:c.1723-72_1883+77 | ex9 |
| EXT1 F10 | CACTTGTCATCATGTGATAATGGC | EXT1 R10 | CTGAACCACCAGTGAGTGAAGC | chr8 | 118816912 | 118817187 | *EXT1* | NM_000127.3:c.1884-54_2055+50 | ex10 |
| EXT1 F11 | CTTGCACTTCTCTCATCATTATCC | EXT1 R11 | TGGCTCTGCTGATGAGTGGATC | chr8 | 118811859 | 118812182 | *EXT1* | NM_000127.3:c.2056-46_*92 | ex11 |
| EXT2 F1 | GGCATGAGCCGGTGACCAAGC | EXT2 R1 | ACGCCCCCGACCACCCAGTC | chr11 | 44117350 | 44117924 | *EXT2* | NM_207122.2:c.-82_-31+523 | ex1 |
| EXT2 F2 | CTTTTCAAGTGTCATTTGCCATCC | EXT2 R2 | TGAGGGCCACTCAAGTATCTCC | chr11 | 44129169 | 44129850 | *EXT2* | NM_207122.2:c.-30-64_536+52 | ex2 |
| EXT2 F3 | GTTGTCTAGTAACTGACTCTTGTC | EXT2 R3 | ATGAACAAAATGATCTTGAACCCATC | chr11 | 44130671 | 44130888 | *EXT2* | NM_207122.2:c.537-71_626+57 | ex3 |
| EXT2 F4 | TGTTCCTCTCCACAGTGTGTATC | EXT2 R4 | ATCTCACAGATTCAGTAAAGGCAC | chr11 | 44135662 | 44135901 | *EXT2* | NM_207122.2:c.627-73_743+50 | ex4 |
| EXT2 F5 | GTTTGTCTTACCTTGACTAACATAC | EXT2 R5 | TTTTAACCCATGTAAGCAAACTCTC | chr11 | 44146274 | 44146594 | *EXT2* | NM_207122.2:c.744-65_939+60 | ex5 |
| EXT2 F6 | TGTAGGGATCAAAGTTAGTGGATC | EXT2 R6 | ACCAGGGTATACATTACAATGACC | chr11 | 44148298 | 44148596 | *EXT2* | NM_207122.2:c.940-67_1079+92 | ex6 |
| EXT2 F7 | TTCTGCTTGTGAAATGAAACAAGAC | EXT2 R7 | CCATTTCGGTACCACCCATGTC | chr11 | 44151506 | 44151735 | *EXT2* | NM_207122.2:c.1080-88_1173+48 | ex7 |
| EXT2 F8 | CACTCTGTCTCGCTTGCTCAC | EXT2 R8 | ACCATGCCTCATGTGGCTAGC | chr11 | 44193114 | 44193329 | *EXT2* | NM_207122.2:c.1174-46_1305+38 | ex8 |
| EXT2 F9 | CATATTGTTACAGCTGCTTTTCTGAC | EXT2 R9 | CAAAGGATGTGAATAATTTGGCCATC | chr11 | 44219329 | 44219634 | *EXT2* | NM_207122.2:c.1306-50_1495+66 | ex9 |
| EXT2 F10 | TGCTTTTACTACTTTATCTCCTCAC | EXT2 R10 | GCAGTCATAGGAAGTAATATTAAAC | chr11 | 44228277 | 44228564 | *EXT2* | NM_207122.2:c.1496-65_1662+56 | ex10 |
| EXT2 F11 | TCAGCACTGAATGGTTGCTGTC | EXT2 R11 | ATTCTCTCAGTTTTGTCACCTTGC | chr11 | 44253842 | 44254089 | *EXT2* | NM_207122.2:c.1663-61_1806+43 | ex11 |
| EXT2 F12 | CTTATTTATCAGCTAAAGGGAACTGC | EXT2 R12 | TAGTTACAAGAACTTCCTAGGCTC | chr11 | 44255601 | 44255872 | *EXT2* | NM_207122.2:c.1807-64_1935+79 | ex12 |
| EXT2 F13 | TTTATTGTCCTTGACACTGACAGC | EXT2 R13 | AGGAAATAGAGATCAGAGGCTAAG | chr11 | 44257791 | 44257977 | *EXT2* | NM_207122.2:c.1936-52_2018+52 | ex13 |
| EXT2 F14 | CCCCATCCTTCTCATTCTGCTC | EXT2 R14 | CCTTAACCTACTACTCTGACATC | chr11 | 44265640 | 44265930 | *EXT2* | NM_207122.2:c.2019-59_*93 | ex14 |
