# Supplementary material for: The Missing Piece of the Puzzle: Unveiling the Role of PTPN11 Gene in Multiple Osteochondromas in a Large Cohort Study
Source: Hum Mutat. 2024 Feb 12;2024:8849348. doi: 10.1155/2024/8849348 (PMC11918999; doi:10.1155/2024/8849348)
Supplement: Supplementary 2 — Table S2: clinical and molecular data of 244 probands from unrelated families. [file 8849348.f2.docx]

**Suppl. Table S2. Clinical and molecular data of 244 probands from unrelated families. Part A. Molecular findings.**

| **Patient #** | **Inh** | **Sex** | **Perfomed testss** | **Gene** | **HGVS_Genomic_GRCh37** | **HGVS_transcript** | **HGVS_Predicted_Protein** | **Type** | **Segregation results** | **exon/**  **intron #** | **Recurrent in our cohort** | **ACMG classes** | **ACMG criteria** | **HGMD v.2022.1 ID #** |
| --- | --- | --- | --- | --- | --- | --- | --- | --- | --- | --- | --- | --- | --- | --- |
| EXT-189 | F | M | Gene panel, MLPA | *EXT2* | chr11:(44117182_44117797)_(44117849_44125021)del | NM_207122.2:c.(-309_-31+395)_(-31+448_-30-4212)del | NP_997005.1:p.? | gross deletion | no data | ex1 |  | PAT | PM2, PVS1, PP5, PP4 | CG211774 |
| EXT-16 | S | F | Sanger EXT1/2, MLPA | *EXT2* | chr11:(44117182_44117797)_(44125084_44129398)del | NM_207122.2:c.(-309_-31+395)_(-30-4212_136)del | NP_997005.1:p.? | gross deletion | no data | ex1 |  | PAT | PM2, PVS1, PP5, PP4 | CG211774 |
| EXT_ND_3 | ND | M | Sanger EXT1/2, MLPA | *EXT2* | chr11:(44125084_44129398)_(44265771_?)del | NM_207122.2:c.(-30-4149_136)_(2091_?)del | NP_997005.1:p.? | gross deletion | no data | ex2-ex14 |  | LPAT | PM2, PVS1 | novel |
| EXT-83 | F | F | Sanger EXT1/2, MLPA | *EXT2* | chr11:(44135796_44146454)_(44148548_44151581)del | NM_207122.2:c.(688_859)_(1079+43_1080-13)del | NP_997005.1:p.? | gross deletion | no data | ex5-ex6 |  | VUS | PM2, PM4, PP4 | novel |
| EXT-131 | S | M | Sanger EXT1/2, MLPA | *EXT2* | chr11:(44151648_44193218)_(44193291_44219412)del | NM_207122.2:c.(1133_1231)_(1304_1339)del | NP_997005.1:p.? | gross deletion | no data | ex8 | yes | PAT | PM2, PVS1, PP5, PP4 | CG099387 |
| EXT-79 | F | M | Sanger EXT1/2, MLPA | *EXT2* | chr11:(44151648_44193218)_(44193291_44219412)del | NM_207122.2:c.(1133_1231)_(1304_1339)del | NP_997005.1:p.? | gross deletion | no data | ex8 | yes | PAT | PM2, PVS1, PP5, PP4 | CG099387 |
| EXT-155 | F | F | Sanger EXT1/2, MLPA | *EXT2* | chr11:(44151648_44193218)_(44193291_44219412)del | NM_207122.2:c.(1133_1231)_(1304_1339)del | NP_997005.1:p.? | gross deletion | no data | ex8 | yes | PAT | PM2, PVS1, PP5, PP4 | CG099387 |
| EXT-181 | S | M | Sanger EXT1/2 | *EXT2* | chr11:44129329C>T | NM_207122.2:c.67C>T | NP_997005.1:p.(Arg23Ter) | nonsense | no data | ex2 |  | PAT | PM2, PVS1, PP4, PP5 | CM980644 |
| EXT-73 | F | M | Sanger EXT1/2 | *EXT2* | chr11:44129340T>G | NM_207122.2:c.78T>G | NP_997005.1:p.(Tyr26Ter) | nonsense | no data | ex2 |  | PAT | PM2, PVS1, PP4 | novel |
| EXT-20 | F | M | Gene panel | *EXT2* | chr11:44129615_44129616del | NM_207122.2:c.353_354del | NP_997005.1:p.(Phe118TrpfsTer13) | frameshift | no data | ex2 |  | PAT | PM2, PVS1, PP4 | novel |
| EXT-147 | F | M | Sanger EXT1/2 | *EXT2* | chr11:44129618del | NM_207122.2:c.356del | NP_997005.1:p.(Gly119Alafs151Ter) | frameshift | paternal | ex2 |  | PAT | PM2, PVS1, PP1, PP4 | novel |
| EXT-207 | F | M | Gene panel | *EXT2* | chr11:44129691C>A | NM_207122.2:c.429C>A | NP_997005.1:p.(Tyr143Ter) | nonsense | paternal | ex2 | yes | PAT | PM2, PVS1, PP4, PP5 | novel |
| EXT-5 | S | F | Sanger EXT1/2 | *EXT2* | chr11:44129691C>A | NM_207122.2:c.429C>A | NP_997005.1:p.(Tyr143Ter) | nonsense | no data | ex2 | yes | PAT | PM2, PVS1, PP4, PP5 | novel |
| EXT-165 | F | M | Sanger EXT1/2 | *EXT2* | chr11:44129691C>A | NM_207122.2:c.429C>A | NP_997005.1:p.(Tyr143Ter) | nonsense | no data | ex2 | yes | PAT | PM2, PVS1, PP4, PP5 | novel |
| EXT-58 | S | M | Sanger EXT1/2 | *EXT2* | chr11:44129717_44129719delinsCGTC | NM_207122.2:c.455_457delinsCGTC | NP_997005.1:p.(Leu152ProfsTer10) | frameshift | de novo | ex2 |  | PAT | PM2, PVS1, PS2, PP4 | novel |
| EXT-2 | S | F | Sanger EXT1/2 | *EXT2* | chr11:44129717T>C | NM_207122.2:c.455T>C | NP_997005.1:p.(Leu152Pro) | missense | de novo | ex2 |  | PAT | PM2, PM5, PP3, PS2, PP4 | novel |
| EXT-38 | S | F | Sanger EXT1/2 | *EXT2* | chr11:44129776C>T | NM_207122.2:c.514C>T | NP_997005.1:p.(Gln172Ter) | nonsense | no data | ex2 | yes | PAT | PM2, PVS1, PP4, PP5 | CM960517 |
| EXT-208 | S | M | Gene panel | *EXT2* | chr11:44129776C>T | NM_207122.2:c.514C>T | NP_997005.1:p.(Gln172Ter) | nonsense | de novo | ex2 | yes | PAT | PM2, PVS1, PS2, PP4, PP5 | CM960517 |
| EXT-67 | F | M | Sanger EXT1/2 | *EXT2* | chr11:44129799G>A | NM_207122.2:c.536+1G>A | NP_997005.1:p.? | splicing | no data | int2 | yes | PAT | PM2, PVS1, PP4, PP5 | CS000222 |
| EXT-140 | F | M | Sanger EXT1/2 | *EXT2* | chr11:44129799G>A | NM_207122.2:c.536+1G>A | NP_997005.1:p.? | splicing | no data | int2 | yes | PAT | PM2, PVS1, PP4, PP5 | CS000222 |
| EXT-18 | F | M | Gene panel | *EXT2* | chr11:44129799G>T | NM_207122.2:c.536+1G>T | NP_997005.1:p.? | splicing | no data | int2 | yes | PAT | PM2, PVS1, PP4 | novel |
| EXT-10 | F | M | Sanger EXT1/2 | *EXT2* | chr11:44129799G>T | NM_207122.2:c.536+1G>T | NP_997005.1:p.? | splicing | no data | int2 | yes | PAT | PM2, PVS1, PP4 | novel |
| EXT-111 | S | M | Sanger EXT1/2 | *EXT2* | chr11:44130743G>A | NM_207122.2:c.537-1G>A | NP_997005.1:p.? | splicing | no data | int2 |  | PAT | PM2, PVS1, PP4, PP5 | CS010544 |
| EXT-166 | F | M | Sanger EXT1/2 | *EXT2* | chr11:44130751C>T | NM_207122.2:c.544C>T | NP_997005.1:p.(Arg182Ter) | nonsense | no data | ex3 |  | PAT | PM2, PVS1, PP4, PP5 | CM000143 |
| EXT-120 | F | M | Sanger EXT1/2 | *EXT2* | chr11:44130814del | NM_207122.2:c.607del | NP_997005.1:p.(Leu203TrpfsTer67) | frameshift | no data | ex3 |  | PAT | PM2, PVS1, PP4, PP5 | CD072408 |
| EXT-193 | F | F | Gene panel | *EXT2* | chr11:44130834_44130837del | NM_207122.2:c.626+1_626+4del | NP_997005.1:p.? | splicing | no data | int3 |  | PAT | PM2, PVS1, PP4 | novel |
| EXT-81 | F | M | Sanger EXT1/2 | *EXT2* | chr11:44130835_44130837del | NM_207122.2:c.626+2_626+4del | NP_997005.1:p.? | splicing | no data | int3 | yes | PAT | PM2, PVS1, PP4 | novel |
| EXT-101 | F | M | Sanger EXT1/2 | *EXT2* | chr11:44130835_44130837del | NM_207122.2:c.626+2_626+4del | NP_997005.1:p.? | splicing | no data | int3 | yes | PAT | PM2, PVS1, PP4 | novel |
| EXT-164 | F | F | Sanger EXT1/2 | *EXT2* | chr11:44130835_44130838del | NM_207122.2:c.626+2_626+5del | NP_997005.1:p.? | splicing | no data | int3 |  | PAT | PM2, PVS1, PP4, PP5 | CD1717261 |
| EXT-7 | F | M | Sanger EXT1/2 | *EXT2* | chr11:44130835T>G | NM_207122.2:c.626+2T>G | NP_997005.1:p.? | splicing | no data | int3 | yes | PAT | PM2, PVS1, PP4 | novel |
| EXT-93 | F | F | Sanger EXT1/2 | *EXT2* | chr11:44130835T>G | NM_207122.2:c.626+2T>G | NP_997005.1:p.? | splicing | no data | int3 | yes | PAT | PM2, PVS1, PP4 | novel |
| EXT-65 | S | F | Gene panel | *EXT2* | chr11:44135760del | NM_207122.2:c.652del | NP_997005.1:p.(Ser218LeufsTer52) | frameshift | no data | ex4 |  | PAT | PM2, PVS1, PP4 | novel |
| EXT-206 | F | F | Sanger EXT1/2 | *EXT2* | chr11:44135786C>A | NM_207122.2:c.678C>A | NP_997005.1:p.(Tyr226Ter) | nonsense | no data | ex4 |  | PAT | PM2, PVS1, PP4 | novel |
| EXT-22 | F | M | Sanger EXT1/2 | *EXT2* | chr11:44135787G>A | NM_207122.2:c.679G>A | NP_997005.1:p.(Asp227Asn) | missense | no data | ex4 |  | LPAT | PM2, PM5, PP3, PM1, PP5, PP4 | CM970453 |
| EXT-173 | F | M | Gene panel | *EXT2* | chr11:44135852G>A | NM_207122.2:c.743+1G>A | NP_997005.1:p.? | splicing | maternal | int4 |  | PAT | PM2, PVS1, PP1, PP5, PP4 | CS050795 |
| EXT_ND_8 | ND | M | Sanger EXT1/2 | *EXT2* | chr11:44146338G>A | NM_207122.2: c.744-1G>A | NP_997005.1:p.? | splicing | no data | int4 |  | LPAT | PM2, PVS1 | novel |
| EXT-122 | S | F | Sanger EXT1/2 | *EXT2* | chr11:44146370dup | NM_207122.2:c.775dup | NP_997005.1:p.(Val259GlyfsTer6) | frameshift | no data | ex5 |  | PAT | PM2, PVS1, PP4 | novel |
| EXT-68 | F | F | Sanger EXT1/2 | *EXT2* | chr11:44146478del | NM_207122.2:c.883del | NP_997005.1:p.(Ser295LeufsTer37) | frameshift | no data | ex5 |  | PAT | PM2, PVS1, PP4 | novel |
| EXT-97 | F | F | Gene panel | *EXT2* | chr11:44146536T>G | NM_207122.2:c.939+2T>G | NP_997005.1:p.? | splicing | no data | int5 |  | PAT | PM2, PVS1, PP4, PP5 | CS092524 |
| EXT-128 | S | M | Sanger EXT1/2 | *EXT2* | chr11:44148401del | NM_207122.2:c.975del | NP_997005.1:p.(Leu326TrpfsTer6) | frameshift | no data | ex6 |  | PAT | PM2, PVS1, PP4 | novel |
| EXT-194 | S | M | Gene panel | *EXT2* | chr11:44148430T>C | NM_207122.2:c.1004T>C | NP_997005.1:p.(Leu335Ser) | missense | no data | ex6 |  | VUS | PM2, PP3, PP4 | novel |
| EXT-53 | F | M | Sanger EXT1/2 | *EXT2* | chr11:44148448C>G | NM_207122.2:c.1022C>G | NP_997005.1:p.(Pro341Arg) | missense | no data | ex6 | yes | LPAT | PM2, PM5, PP3, PP4 | novel |
| EXT-1 | F | F | Gene panel | *EXT2* | chr11:44148448C>G | NM_207122.2:c.1022C>G | NP_997005.1:p.(Pro341Arg) | missense | paternal | ex6 | yes | LPAT | PM2, PM5, PP3, PP1, PP4 | novel |
| EXT-162 | F | F | Gene panel | *EXT2* | chr11:44148478C>T | NM_207122.2:c.1052C>T | NP_997005.1:p.(Pro351Leu) | missense | no data | ex6 | yes | LPAT | PM2, PM5, PP3, PM1, PP5, PP4 | CM1910387 |
| EXT-115 | S | M | Sanger EXT1/2 | *EXT2* | chr11:44148478C>T | NM_207122.2:c.1052C>T | NP_997005.1:p.(Pro351Leu) | missense | no data | ex6 | yes | LPAT | PM2, PM5, PP3, PM1, PP5, PP4 | CM1910387 |
| EXT-54 | S | F | Sanger EXT1/2 | *EXT2* | chr11:44148504A>T | NM_207122.2:c.1078A>T | NP_997005.1:p.(Arg360Ter) | nonsense | no data | ex6 |  | PAT | PM2, PVS1, PP4 | novel |
| EXT-190 | F | F | Gene panel | *EXT2* | chr11:44148506G>T | NM_207122.2:c.1079+1G>T | NP_997005.1:p.? | splicing | no data | int6 | yes | PAT | PM2, PVS1, PP4, PP5 | CS982174 |
| EXT-47 | F | M | Sanger EXT1/2 | *EXT2* | chr11:44148506G>T | NM_207122.2:c.1079+1G>T | NP_997005.1:p.? | splicing | no data | int6 | yes | PAT | PM2, PVS1, PP4, PP5 | CS982174 |
| EXT-84 | ND | F | Gene panel | *EXT2* | chr11:44151647C>T | NM_207122.2:c.1132C>T | NP_997005.1:p.(Gln378Ter) | nonsense | no data | ex7 |  | PAT | PM2, PVS1, PP4, PP5 | CM000512 |
| EXT-64 | S | F | Gene panel | *EXT2* | chr11:44151680C>T | NM_207122.2:c.1165C>T | NP_997005.1:p.(Gln389Ter) | nonsense | no data | ex7 |  | PAT | PM2, PVS1, PP4 | novel |
| EXT-209 | F | F | Gene panel | *EXT2* | chr11:44151688_44151689insAT | NM_207122.2:c.1173_1173+1insAT | NP_997005.1:p.? | splicing | no data | int7 |  | PAT | PM2, PVS1, PP4 | novel |
| EXT-195 | S | F | Gene panel | *EXT2* | chr11:44151689G>A | NM_207122.2:c.1173+1G>A | NP_997005.1:p.? | splicing | no data | int7 |  | PAT | PM2, PVS1, PP4, PP5 | CS961558 |
| EXT-36 | F | M | Gene panel | *EXT2* | chr11:44193168G>A | NM_207122.2:c.1181G>A | NP_997005.1:p.(Trp394Ter) | nonsense | no data | ex8 | yes | PAT | PM2, PVS1, PP4, PP5 | CM149867 |
| EXT-49 | F | M | Gene panel | *EXT2* | chr11:44193168G>A | NM_207122.2:c.1181G>A | NP_997005.1:p.(Trp394Ter) | nonsense | no data | ex8 | yes | PAT | PM2, PVS1, PP4, PP5 | CM149867 |
| EXT-172 | S | F | Sanger EXT1/2, MLPA, gene panel | *PTPN11* | chr12:112888307del | NM_002834.5:c.324del | NP_002825.3:p.(Ser109LeufsTer18) | frameshift | de novo | ex3 |  | PAT | PM2, PVS1, PS3, PS2, PP4 | novel |
| EXT-151 | F | F | Sanger EXT1/2, MLPA, Noonan gene panel | *PTPN11* | chr12:112891184_112891185del | NM_002834.5:c.518_519del | NP_002825.3:p.(Arg173LeufsTer15) | frameshift | paternal | ex4 |  | PAT | PM2, PVS1, PP1, PP4 | novel |
| EXT-205 | S | M | Sanger EXT1/2, MLPA, gene panel | *PTPN11* | chr12:112892385del | NM_002834.5:c.543del | NP_002825.3:p.(Gly182ValfsTer9) | frameshift | no data | ex5 |  | PAT | PM2, PVS1, PP4 | novel |
| EXT-57 | S | M | Gene panel | *PTPN11* | chr12:112892431del | NM_002834.5:c.589del | NP_002825.3:p.(Tyr197IlefsTer25) | frameshift | no data | ex5 | yes | PAT | PM2, PVS1, PP4 | novel |
| EXT-13 | S | M | Sanger EXT1/2, MLPA, Noonan gene panel | *PTPN11* | chr12:112892431del | NM_002834.5:c.589del | NP_002825.3:p.(Tyr197IlefsTer25) | frameshift | de novo | ex5 | yes | PAT | PM2, PVS1, PS2, PP4 | novel |
| EXT-35 | S | M | Noonan gene panel | *PTPN11* | chr12:112910760C>T | NM_002834.5:c.769C>T | NP_002825.3:p.(Gln257Ter) | nonsense | de novo | ex7 |  | PAT | PM2, PVS1, PS2, PP4 | novel |
| EXT-184 | S | M | Gene panel | *PTPN11* | chr12:112915536dup | NM_002834.5:c.933+2dup | NP_002825.3:p.? | splicing | de novo | int 8 |  | PAT | PM2, PVS1, PS3, PS2, PP4 | novel |
| EXT-170 | F | M | Sanger EXT1/2, MLPA, gene panel | *PTPN11* | chr12:112919979_112919980del | NM_002834.5:c.1194_1195del | NP_002825.3:p.(Glu400ThrfsTer2) | frameshift | maternal | ex10 |  | PAT | PM2, PVS1, PP1, PP4 | novel |
| EXT-210 | S | F | Gene panel | *PTPN11* | chr12:112926943T>A | NM_002834.5:c.1563T>A | NP_002825.3:p.(Tyr521Ter) | nonsense | de novo | ex13 |  | PAT | PM2, PVS1, PS2, PP4 | novel |
| EXT-75 | S | M | Gene panel, MLPA | *EXT1* | chr8:(?_118811842)_(118811902_118849259)del | NM_000127.3:c.(720_1056+22)_(*109_?)del | NP_000118.2:p.? | gross deletion | no data | ex2-ex11 | yes | PAT | PM2, PVS1, PP5, PP4 | CG1815021 |
| EXT-211 | S | M | Gene panel, MLPA | *EXT1* | chr8:(?_118811842)_(118811902_118849259)del | NM_000127.3:c.(720_1056+22)_(*109_?)del | NP_000118.2:p.? | gross deletion | no data | ex2-ex11 | yes | PAT | PM2, PVS1, PP5, PP4 | CG1815021 |
| EXT-117 | S | M | Sanger EXT1/2, MLPA | *EXT1* | chr8:(?_118811842)_(118811902_118849259)del | NM_000127.3:c.(720_1056+22)_(*109_?)del | NP_000118.2:p.? | gross deletion | no data | ex2-ex11 | yes | PAT | PM2, PVS1, PP5, PP4 | CG1815021 |
| EXT-87 | F | F | Sanger EXT1/2, MLPA | *EXT1* | chr8:(?_118811842)_(118811902_118849259)del | NM_000127.3:c.(720_1056+22)_(*109_?)del | NP_000118.2:p.? | gross deletion | no data | ex2-ex11 | yes | PAT | PM2, PVS1, PP5, PP4 | CG1815021 |
| EXT_ND_12 | ND | M | Sanger EXT1/2, MLPA | *EXT1* | chr8:(?_118811842)_(118817096_118819531)del | NM_000127.3:c.(1881_1920)_(*109_?)del | NP_000118.2:p.? | gross deletion | no data | ex10-ex11 |  | VUS | PM2, PM4 | novel |
| EXT-107 | F | F | Gene panel, MLPA | *EXT1* | chr8:(?_118811842)_(119123291_?)del | NM_000127.3:c.(?_-5)_(*109_?)del | NP_000118.2:p.? | gross deletion | no data | ex1-ex11 |  | PAT | PM2, PVS1, PP5, PP4 | CG024879 |
| EXT-212 | S | M | Gene panel, MLPA | *EXT1* | chr8:(118812094_118817029)_(118819531_118825129)del | NM_000127.3:c.(1704_1808)_(1987_2098)del | NP_000118.2:p.? | gross deletion | no data | ex9-ex10 |  | PAT | PM2, PVS1, PP5, PP4 | novel |
| EXT-183 | S | F | Gene panel, MLPA | *EXT1* | chr8:(118831982_118834697)_(118834776_118842473)del | NM_000127.3:c.(1286_1345)_(1417+7_1469)del | NP_000118.2:p.? | gross deletion | no data | ex5 |  | PAT | PM2, PVS1, PP5, PP4 | novel |
| EXT-186 | S | F | Sanger EXT1/2, MLPA | *EXT1* | chr8:(118834776_118842473)_(118849259_119122566)dup | NM_000127.3:c.(720_1056+22)_(1286_1345)dup | NP_000118.2:p.? | gross duplication | paternal | ex2-ex4 |  | LPAT | PM2, PM4, PP1, PP4 | novel |
| EXT-95 | F | F | Sanger EXT1/2, MLPA | *EXT1* | chr8:(119122566_119123222)_119123291_?)del | NM_000127.3:c.(?_-5)_(720_1056+22)del | NP_000118.2:p.? | gross deletion | no data | ex1 |  | PAT | PM2, PVS1, PP5, PP4 | CG044328 |
| EXT-69 | S | M | Gene panel | *EXT1* | chr8:118812060C>T | NM_000127.3:c.2132G>A | NP_000118.2:p.(Trp711Ter) | nonsense | no data | ex11 | yes | LPAT | PM2, PM4, PP3, PP5, PP4 | CM1821677 |
| EXT-30 | F | M | Gene panel | *EXT1* | chr8:118812060C>T | NM_000127.3:c.2132G>A | NP_000118.2:p.(Trp711Ter) | nonsense | no data | ex11 | yes | LPAT | PM2, PM4, PP3, PP5, PP4 | CM1821677 |
| EXT-103 | F | F | Gene panel | *EXT1* | chr8:118812091G>A | NM_000127.3:c.2101C>T | NP_000118.2:p.(Arg701Ter) | nonsense | no data | ex11 | yes | LPAT | PM2, PM4, PP3, PP5, PP4 | CM010236 |
| EXT-77 | S | M | Sanger EXT1 | *EXT1* | chr8:118812091G>A | NM_000127.3:c.2101C>T | NP_000118.2:p.(Arg701Ter) | nonsense | no data | ex11 | yes | LPAT | PM2, PM4, PP3, PP5, PP4 | CM010236 |
| EXT-114 | F | M | Sanger EXT1 | *EXT1* | chr8:118812116C>T | NM_000127.3:c.2076G>A | NP_000118.2:p.(Trp692Ter) | nonsense | no data | ex11 |  | LPAT | PM2, PM4, PP3, PP5, PP4 | CM099185 |
| EXT-126 | S | M | Sanger EXT1 | *EXT1* | chr8:118812123del | NM_000127.3:c.2071del | NP_000118.2:p.(Arg691ValfsTer15) | frameshift | no data | ex11 |  | PAT | PM2, PM4, PP3, PP5, PP4 | novel |
| EXT_ND_13 | ND | M | Sanger EXT1 | *EXT1* | chr8:118816959del | NM_000127.3:c.2055+2del | NP_000118.2:p.? | splicing | no data | int10 |  | LPAT | PM2, PVS1 | novel |
| EXT-92 | F | F | Gene panel | *EXT1* | chr8:118817020T>A | NM_000127.3:c.1996A>T | NP_000118.2:p.(Lys666Ter) | nonsense | no data | ex10 |  | PAT | PM2, PVS1, PP4 | novel |
| EXT-23 | S | F | Sanger EXT1 | *EXT1* | chr8:118817097del | NM_000127.3:c.1920del | NP_000118.2:p.(Ser641AlafsTer2) | frameshift | no data | ex10 |  | PAT | PM2, PVS1, PP4 | novel |
| EXT-52 | F | M | Sanger EXT1 | *EXT1* | chr8:118817133C>G | NM_000127.3:c.1884-1G>C | NP_000118.2:p.? | splicing | no data | int9 |  | PAT | PM2, PVS1, PP3, PP4,PP5 | CS1413639 |
| EXT-56 | F | M | Sanger EXT1 | *EXT1* | chr8:118817134T>C | NM_000127.3:c.1884-2A>G | NP_000118.2:p.? | splicing | no data | int9 | yes | PAT | PM2, PVS1, PP3, PP4,PP5 | CS189262 |
| EXT-192 | F | F | Sanger EXT1 | *EXT1* | chr8:118817134T>C | NM_000127.3:c.1884-2A>G | NP_000118.2:p.? | splicing | no data | int9 | yes | PAT | PM2, PVS1, PP3, PP4,PP5 | CS189262 |
| EXT_ND_7 | F | M | Sanger EXT1 | *EXT1* | chr8:118819460_118819462del | NM_000127.3:c.1879_1881del | NP_000118.2:p.(His627del) | in-frame deletion | maternal | ex9 |  | LPAT | PM2, PM4, PP1, PP5 | CD982629 |
| EXT-25 | F | F | Gene panel | *EXT1* | chr8:118819461G>T | NM_000127.3:c.1878C>A | NP_000118.2:p.(Tyr626Ter) | nonsense | no data | ex9 |  | PAT | PM2, PVS1, PP4 | novel |
| EXT-137 | F | F | Sanger EXT1/2 | *EXT1* | chr8:118819504C>T | NM_000127.3:c.1835G>A | NP_000118.2:p.(Trp612Ter) | nonsense | no data | ex9 |  | PAT | PM2, PVS1, PP3, PP4,PP5 | CM099181 |
| EXT-96 | S | M | Gene panel | *EXT1* | chr8:118819563G>T | NM_000127.3:c.1776C>A | NP_000118.2:p.(Tyr592Ter) | nonsense | no data | ex9 |  | PAT | PM2, PVS1, PP3, PP4,PP5 | CM012095 |
| EXT-141 | S | F | Gene panel | *EXT1* | chr8:118819572_118819573del | NM_000127.3:c.1766_1767del | NP_000118.2:p.(Ile589SerfsTer12) | frameshift | de novo | ex9 |  | PAT | PM2, PVS1, PS2, PP4 | novel |
| EXT-109 | S | M | Sanger EXT1 | *EXT1* | chr8:118819617C>T | NM_000127.3:c.1723-1G>A | NP_000118.2:p.? | splicing | de novo | int8 | yes | PAT | PM2, PVS1, PP4, PS2 | novel |
| EXT-177 | F | F | Gene panel | *EXT1* | chr8:118819617C>T | NM_000127.3:c.1723-1G>A | NP_000118.2:p.? | splicing | no data | int8 | yes | PAT | PM2, PVS1, PP4 | novel |
| EXT-21 | F | M | Gene panel | *EXT1* | chr8:118825109A>G | NM_000127.3:c.1722+2T>C | NP_000118.2:p.? | splicing | no data | int8 |  | PAT | PM2, PVS1, PP4 | novel |
| EXT-4 | S | F | Sanger EXT1 | *EXT1* | chr8:118825115_118825128del | NM_000127.3:c.1705_1718del | NP_000118.2:p.(Val569ArgfsTer14) | frameshift | no data | ex8 |  | PAT | PM2, PVS1, PP4 | novel |
| EXT-76 | F | F | Gene panel | *EXT1* | chr8:118825155_118825174dup | NM_000127.3:c.1660_1679dup | NP_000118.2:p.(Val561ThrfsTer67) | frameshift | no data | ex8 |  | PAT | PM2, PVS1, PP4 | novel |
| EXT-130 | S | M | Sanger EXT1 | *EXT1* | chr8:118825191del | NM_000127.3:c.1642del | NP_000118.2:p.(Ser548AlafsTer73) | frameshift | no data | ex8 | yes | PAT | PM2, PVS1, PP3, PP4,PP5 | CD012248 |
| EXT-51 | F | M | Gene panel | *EXT1* | chr8:118825191del | NM_000127.3:c.1642del | NP_000118.2:p.(Ser548AlafsTer73) | frameshift | no data | ex8 | yes | PAT | PM2, PVS1, PP3, PP4,PP5 | CD012248 |
| EXT-124 | S | M | Sanger EXT1 | *EXT1* | chr8:118830672A>C | NM_000127.3:c.1632+2T>G | NP_000118.2:p.? | splicing | no data | int7 |  | PAT | PM2, PVS1, PP4 | novel |
| EXT-203 | F | M | Gene panel | *EXT1* | chr8:118830673C>T | NM_000127.3:c.1632+1G>A | NP_000118.2:p.? | splicing | no data | int7 |  | PAT | PM2, PVS1, PP4 | novel |
| EXT_ND_14 | ND | M | Sanger EXT1 | *EXT1* | chr8:118830741_118830742delinsT | NM_000127.3:c.1564_1565delinsA | NP_000118.2:p.(Pro522ThrfsTer25) | frameshift | no data | ex7 |  | LPAT | PM2, PVS1 | novel |
| EXT-213 | F | M | Gene panel | *EXT1* | chr8:118830770C>A | NM_000127.3:c.1537-1G>T | NP_000118.2:p.? | splicing | no data | int6 |  | PAT | PM2, PVS1, PP4, PP5 | CS050793 |
| EXT-158 | F | F | Sanger EXT1 | *EXT1* | chr8:118830770C>G | NM_000127.3:c.1537-1G>C | NP_000118.2:p.? | splicing | no data | int6 |  | PAT | PM2, PVS1, PP4, PP5 | CS087948 |
| EXT-123 | S | F | Gene panel | *EXT1* | chr8:118831913A>C | NM_000127.3:c.1536+2T>G | NP_000118.2:p.? | splicing | no data | int6 |  | PAT | PM2, PVS1, PP4 | novel |
| EXT-91 | S | M | Sanger EXT1 | *EXT1* | chr8:118831914C>T | NM_000127.3:c.1536+1G>A | NP_000118.2:p.? | splicing | no data | int6 |  | PAT | PM2, PVS1, PP4, PP5 | CS099214 |
| EXT-113 | S | F | Sanger EXT1 | *EXT1* | chr8:118831921del | NM_000127.3:c.1530del | NP_000118.2:p.(Cys510TrpfsTer5) | frameshift | no data | ex6 |  | PAT | PM2, PVS1, PP4 | novel |
| EXT-27 | F | M | Gene panel | *EXT1* | chr8:118831929G>A | NM_000127.3:c.1522C>T | NP_000118.2:p.(Gln508Ter) | nonsense | no data | ex6 | yes | PAT | PM2, PVS1, PP4, PP5 | CM189257 |
| EXT-157 | F | M | Sanger EXT1 | *EXT1* | chr8:118831929G>A | NM_000127.3:c.1522C>T | NP_000118.2:p.(Gln508Ter) | nonsense | no data | ex6 | yes | PAT | PM2, PVS1, PP4, PP5 | CM189257 |
| EXT-179 | F | F | Gene panel | *EXT1* | chr8:118831929G>A | NM_000127.3:c.1522C>T | NP_000118.2:p.(Gln508Ter) | nonsense | maternal | ex6 | yes | PAT | PM2, PVS1, PP4, PP5 | CM189257 |
| EXT-42 | S | F | Sanger EXT1 | *EXT1* | chr8:118831974G>A | NM_000127.3:c.1477C>T | NP_000118.2:p.(Gln493Ter) | nonsense | no data | ex6 |  | PAT | PM2, PVS1, PP4, PP5 | CM087944 |
| EXT-100 | F | M | Gene panel | *EXT1* | chr8:118831982del | NM_000127.3:c.1469del | NP_000118.2:p.(Leu490ArgfsTer9) | frameshift | no data | ex6 | yes | PAT | PM2, PVS1, PP4, PP5 | CD951695 |
| EXT-215 | F | F | Gene panel | *EXT1* | chr8:118831982del | NM_000127.3:c.1469del | NP_000118.2:p.(Leu490ArgfsTer9) | frameshift | no data | ex6 | yes | PAT | PM2, PVS1, PP4, PP5 | CD951695 |
| EXT-214 | S | F | Gene panel | *EXT1* | chr8:118831982del | NM_000127.3:c.1469del | NP_000118.2:p.(Leu490ArgfsTer9) | frameshift | no data | ex6 | yes | PAT | PM2, PVS1, PP4, PP5 | CD951695 |
| EXT-216 | F | F | Gene panel, MLPA | *EXT1* | chr8:118831988del | NM_000127.3:c.1468del | NP_000118.2:p.(Leu490TrpfsTer9) | frameshift | no data | ex6 |  | PAT | PM2, PVS1, PP4 | CD000259 |
| EXT-71 | F | M | Gene panel | *EXT1* | chr8:118831988dup | NM_000127.3:c.1468dup | NP_000118.2:p.(Leu490ProfsTer31) | frameshift | no data | ex6 |  | PAT | PM2, PVS1, PP4, PP5 | CI010703 |
| EXT-40 | S | M | Sanger EXT1 | *EXT1* | chr8:118832019dup | NM_000127.3:c.1432dup | NP_000118.2:p.(Ser478PhefsTer43) | frameshift | no data | ex6 |  | PAT | PM2, PVS1, PP4, PP5 | CI972596 |
| EXT-43 | S | M | Sanger EXT1 | *EXT1* | chr8:118832025del | NM_000127.3:c.1431del | NP_000118.2:p.(Ser478ProfsTer10) | frameshift | no data | ex6 |  | PAT | PM2, PVS1, PP4, PP5 | CD087952 |
| EXT-132 | F | M | Sanger EXT1 | *EXT1* | chr8:118832030A>C | NM_000127.3:c.1421T>G | NP_000118.2:p.(Leu474Ter) | nonsense | no data | ex6 |  | PAT | PM2, PVS1, PP4, PP5 | CM053864 |
| EXT-175 | S | F | Sanger EXT1 | *EXT1* | chr8:118834702del | NM_000127.3:c.1417+2del | NP_000118.2:p.? | splicing | no data | int5 |  | PAT | PM2, PVS1, PP4 | novel |
| EXT-180 | S | M | Sanger EXT1 | *EXT1* | chr8:118834787C>T | NM_000127.3:c.1334G>A | NP_000118.2:p.(Trp445Ter) | nonsense | no data | ex5 |  | PAT | PM2, PVS1, PP4, PP5 | CM189256 |
| EXT_ND_15 | F | F | Sanger EXT1 | *EXT1* | chr8:118834820_118834825del | NM_000127.3:c.1296_1301del | NP_000118.2:p.(Asp432_Ile434delinsGlu) | in-frame deletion | no data | ex5 |  | VUS | PM2, PM4 | novel |
| EXT-217 | F | M | Gene panel | *EXT1* | chr8:118834837C>A | NM_000127.3:c.1285-1G>T | NP_000118.2:p.? | splicing | no data | int4 |  | PAT | PM2, PVS1, PP4 | novel |
| EXT-156 | S | M | Gene panel | *EXT1* | chr8:118842467A>C | NM_000127.3:c.1284+2T>G | NP_000118.2:p.? | splicing | no data | int4 |  | PAT | PM2, PVS1, PP4, PP5 | CS065546 |
| EXT-169 | F | F | Gene panel | *EXT1* | chr8:118842467dup | NM_000127.3:c.1284+2dup | NP_000118.2:p.? | splicing | no data | int4 |  | PAT | PM2, PVS1, PP4 | novel |
| EXT-102 | S | M | Gene panel | *EXT1* | chr8:118842469_118842472delinsG | NM_000127.3:c.1281_1284delinsC | NP_000118.2:p.(Glu428del) | in-frame deletion | de novo | ex4 |  | LPAT | PM2, PM4, PS2, PP4 | novel |
| EXT-118 | S | M | Gene panel | *EXT1* | chr8:118842473A>G | NM_000127.3:c.1280T>C | NP_000118.2:p.(Leu427Pro) | missense | de novo | ex4 |  | LPAT | PM2, PP3, PS2, PP4 | novel |
| EXT-135 | F | M | Sanger EXT1 | *EXT1* | chr8:118842528G>A | NM_000127.3:c.1225C>T | NP_000118.2:p.(Gln409Ter) | nonsense | no data | ex4 | yes | PAT | PM2, PVS1, PP4, PP5 | CM053228 |
| EXT-125 | S | M | Sanger EXT1 | *EXT1* | chr8:118842537_118842540del | NM_000127.3:c.1215_1218del | NP_000118.2:p.(Arg405SerfsTer19) | frameshift | no data | ex4 | yes | PAT | PM2, PVS1, PP4, PP5 | CD982627 |
| EXT-176 | F | M | Sanger EXT1 | *EXT1* | chr8:118842537_118842540del | NM_000127.3:c.1215_1218del | NP_000118.2:p.(Arg405SerfsTer19) | frameshift | no data | ex4 | yes | PAT | PM2, PVS1, PP4, PP5 | CD982627 |
| EXT-66 | F | F | Sanger EXT1 | *EXT1* | chr8:118842537G>A | NM_000127.3:c.1216C>T | NP_000118.2:p.(Gln406Ter) | nonsense | no data | ex4 |  | PAT | PM2, PVS1, PP4, PP5 | M053856 |
| EXT_ND_11 | F | F | Sanger EXT1 | *EXT1* | chr8:118842540dup | NM_000127.3:c.1213dup | NP_000118.2:p.(Arg405LysfsTer16) | frameshift | no data | ex4 |  | PAT | PM2, PVS1, PP5 | CI099373 |
| EXT-17 | S | M | Gene panel | *EXT1* | chr8:118842542_118842548del | NM_000127.3:c.1208_1214del | NP_000118.2:p.(Ala403AspfsTer20) | frameshift | no data | ex4 |  | PAT | PM2, PVS1, PP4 | novel |
| EXT_ND_16 | ND | M | Sanger EXT1 | *EXT1* | chr8:118842561G>A | NM_000127.3:c.1192C>T | NP_000118.2:p.(Gln398Ter) | nonsense | no data | ex4 |  | PAT | PM2, PVS1, PP5 | CM065161 |
| EXT-14 | F | M | Sanger EXT1 | *EXT1* | chr8:118842583del | NM_000127.3:c.1171del | NP_000118.2:p.(Ser391LeufsTer12) | frameshift | no data | ex4 |  | PAT | PM2, PVS1, PP4, PP5 | CD072400 |
| EXT-178 | F | F | Sanger EXT1 | *EXT1* | chr8:118847685_118847697del | NM_000127.3:c.1152_1164del | NP_000118.2:p.(Leu385PhefsTer14) | frameshift | no data | ex3 |  | PAT | PM2, PVS1, PP4 | novel |
| EXT-74 | S | M | Sanger EXT1 | *EXT1* | chr8:118847685G>A | NM_000127.3:c.1162C>T | NP_000118.2:p.(Gln388Ter) | nonsense | no data | ex3 |  | PAT | PM2, PVS1, PP4, PP5 | CM053855 |
| EXT-138 | S | M | Gene panel | *EXT1* | chr8:118847696del | NM_000127.3:c.1151del | NP_000118.2:p.(Arg384AsnfsTer19) | frameshift | de novo | ex3 |  | PAT | PM2, PVS1, PS2, PP4 | novel |
| EXT-108 | S | M | Sanger EXT1 | *EXT1* | chr8:118847718C>T | NM_000127.3:c.1129G>A | NP_000118.2:p.(Ala377Thr) | missense | no data | ex3 |  | LPAT | PM2, PM5, PP3, PP4 | novel |
| EXT-33 | F | M | Gene panel | *EXT1* | chr8:118847721G>A | NM_000127.3:c.1126C>T | NP_000118.2:p.(Gln376Ter) | nonsense | no data | ex3 |  | PAT | PM2, PVS1, PP4,PP5 | CM099169 |
| EXT-196 | S | F | Gene panel | *EXT1* | chr8:118847737del | NM_000127.3:c.1110delA | NP_000118.2:p.(Val371Ter) | nonsense | no data | ex3 |  | PAT | PM2, PVS1, PP4 | novel |
| EXT-161 | S | F | Sanger EXT1 | *EXT1* | chr8:118847754C>A | NM_000127.3:c.1093G>T | NP_000118.2:p.(Glu365Ter) | nonsense | no data | ex3 |  | PAT | PM2, PVS1, PP4 | novel |
| EXT-39 | S | F | Sanger EXT1 | *EXT1* | chr8:118847774del | NM_000127.3:c.1073del | NP_000118.2:p.(Val358GlyfsTer14) | frameshift | no data | ex3 |  | PAT | PM2, PVS1, PP4 | novel |
| EXT-153 | F | M | Sanger EXT1 | *EXT1* | chr8:118847779del | NM_000127.3:c.1070del | NP_000118.2:p.(Pro357LeufsTer2) | frameshift | no data | ex3 |  | PAT | PM2, PVS1, PP4 | novel |
| EXT-3 | F | F | Sanger EXT1 | *EXT1* | chr8:118847792T>C | NM_000127.3:c.1057-2A>G | NP_000118.2:p.? | splicing | maternal | int2 |  | PAT | PM2, PVS1, PP1, PP5, PP4 | CS982173 |
| EXT-142 | S | F | Sanger EXT1 | *EXT1* | chr8:118847793G>C | NM_000127.3:c.1057-3C>G | NP_000118.2:p.? | splicing | no data | int2 |  | PAT | PM2, PVS1, PP4, PP5 | CS099210 |
| EXT-218 | F | F | Gene panel | *EXT1* | chr8:118849346C>T | NM_000127.3:c.1056+1G>A | NP_000118.2:p.? | splicing | no data | int2 | yes | PAT | PM2, PVS1, PP4, PP5 | CS971723 |
| EXT-163 | S | F | Gene panel | *EXT1* | chr8:118849346C>T | NM_000127.3:c.1056+1G>A | NP_000118.2:p.? | splicing | no data | int2 | yes | PAT | PM2, PVS1, PP4, PP5 | CS971723 |
| EXT_ND_6 | ND | M | Sanger EXT1 | *EXT1* | chr8:118849367T>A | NM_000127.3:c.1036A>T | NP_000118.2:p.(Arg346Ter) | nonsense | no data | ex2 |  | LPAT | PM2, PVS1 | novel |
| EXT_ND_17 | ND | M | Sanger EXT1 | *EXT1* | chr8:118849367T>C | NM_000127.3:c.1036A>G | NP_000118.2:p.(Arg346Gly) | missense | no data | ex2 |  | LPAT | PM2, PM5, PP3, PM1 | CM071716 |
| EXT-60 | F | M | Sanger EXT1 | *EXT1* | chr8:118849372G>T | NM_000127.3:c.1031C>A | NP_000118.2:p.(Ser344Tyr) | missense | no data | ex2 |  | LPAT | PM2, PM5, PP3, PP4 | novel |
| EXT-154 | S | M | Sanger EXT1 | *EXT1* | chr8:118849384C>A | NM_000127.3:c.1019G>T | NP_000118.2:p.(Arg340Leu) | missense | no data | ex2 | yes | PAT | PM2, PM5, PP3, PS3, PM1, PP4 | CM970448 |
| EXT-187 | F | M | Sanger EXT1 | *EXT1* | chr8:118849384C>A | NM_000127.3:c.1019G>T | NP_000118.2:p.(Arg340Leu) | missense | no data | ex2 | yes | PAT | PM2, PM5, PP3, PS3, PM1, PP4 | CM970448 |
| EXT-121 | F | M | Gene panel | *EXT1* | chr8:118849384C>A | NM_000127.3:c.1019G>T | NP_000118.2:p.(Arg340Leu) | missense | no data | ex2 | yes | PAT | PM2, PM5, PP3, PS3, PM1, PP4 | CM970448 |
| EXT-143 | F | M | Sanger EXT1 | *EXT1* | chr8:118849384C>A | NM_000127.3:c.1019G>T | NP_000118.2:p.(Arg340Leu) | missense | no data | ex2 | yes | PAT | PM2, PM5, PP3, PS3, PM1, PP4 | CM970448 |
| EXT-59 | F | M | Sanger EXT1 | *EXT1* | chr8:118849384C>A | NM_000127.3:c.1019G>T | NP_000118.2:p.(Arg340Leu) | missense | maternal | ex2 | yes | PAT | PM2, PM5, PP3, PS3, PM1, PP4 | CM970448 |
| EXT-98 | F | F | Sanger EXT1 | *EXT1* | chr8:118849385G>A | NM_000127.3:c.1018C>T | NP_000118.2:p.(Arg340Cys) | missense | no data | ex2 |  | PAT | PM2, PM5, PP3, PS3, PM1, PP4 | CM970449 |
| EXT-6 | S | M | Gene panel | *EXT1* | chr8:118849387C>A | NM_000127.3:c.1016G>T | NP_000118.2:p.(Gly339Val) | missense | no data | ex2 |  | LPAT | PM2, PM5, PP3, PM1, PP5, PP5 | CM053233 |
| EXT-127 | F | M | Gene panel | *EXT1* | chr8:118849387C>T | NM_000127.3:c.1016G>A | NP_000118.2:p.(Gly339Asp) | missense | maternal | ex2 |  | LPAT | PM2, PM5, PP3, PM1, PP5, PP5 | CM970447 |
| EXT-219 | F | F | Gene panel | *EXT1* | chr8:118849388C>G | NM_000127.3:c.1015G>C | NP_000118.2:p.(Gly339Arg) | missense | no data | ex2 |  | LPAT | PM2, PS1, PP3, PP4 | novel |
| EXT-94 | S | M | Gene panel | *EXT1* | chr8:118849404_118849405del | NM_000127.3:c.999_1000del | NP_000118.2:p.(Phe333LeufsTer37) | frameshift | no data | ex2 |  | PAT | PM2, PVS1, PP4 | novel |
| EXT-106 | F | F | Sanger EXT1 | *EXT1* | chr8:118849441C>G | NM_000127.3:c.963-1G>C | NP_000118.2:p.? | splicing | no data | int1 |  | PAT | PM2, PVS1, PP4 | novel |
| EXT_ND_18 | ND | F | Sanger EXT1 | *EXT1* | chr8:118849442T>A | NM_000127.3:c.963-2A>T | NP_000118.2:p.? | splicing | no data | int1 |  | LPAT | PM2, PVS1 | novel |
| EXT-197 | F | M | Gene panel | *EXT1* | chr8:119122323C>T | NM_000127.3:c.962+1G>A | NP_000118.2:p.? | splicing | no data | int1 |  | PAT | PM2, PVS1, PP4, PP5 | CS099207 |
| EXT-44 | F | F | Gene panel | *EXT1* | chr8:119122345_119122346del | NM_000127.3:c.943_944del | NP_000118.2:p.(Asp315GlnfsTer5) | frameshift | maternal | ex1 |  | PAT | PM2, PVS1, PP4 | CD000604 |
| EXT-37 | F | F | Sanger EXT1 | *EXT1* | chr8:119122374C>T | NM_000127.3:c.912G>A | NP_000118.2:p.(Trp304Ter) | nonsense | no data | ex1 |  | PAT | PM2, PVS1, PP4, PP5 | CM099157 |
| EXT-78 | F | F | Gene panel | *EXT1* | chr8:119122375C>T | NM_000127.3:c.911G>A | NP_000118.2:p.(Trp304Ter) | nonsense | no data | ex1 |  | PAT | PM2, PVS1, PP4 | novel |
| EXT-152 | F | M | Gene panel | *EXT1* | chr8:119122379del | NM_000127.3:c.907del | NP_000118.2:p.(Asp303ThrfsTer56) | frameshift | no data | ex1 |  | PAT | PM2, PVS1, PP4 | novel |
| EXT_ND_10 | F | M | Sanger EXT1 | *EXT1* | chr8:119122391T>A | NM_000127.3:c.895A>T | NP_000118.2:p.(Lys299Ter) | nonsense | no data | ex1 |  | LPAT | PM2, PVS1 | novel |
| EXT-34 | F | M | Gene panel | *EXT1* | chr8:119122446C>G | NM_000127.3:c.840G>C | NP_000118.2:p.(Arg280Ser) | missense | no data | ex1 | yes | LPAT | PM2, PM5, PP3, PM1, PP5, PP5 | CM980638 |
| EXT-198 | ND | M | Gene panel | *EXT1* | chr8:119122446C>G | NM_000127.3:c.840G>C | NP_000118.2:p.(Arg280Ser) | missense | no data | ex1 | yes | LPAT | PM2, PM5, PP3, PM1, PP5, PP5 | CM980638 |
| EXT-144 | F | M | Sanger EXT1 | *EXT1* | chr8:119122447C>A | NM_000127.3:c.839G>T | NP_000118.2:p.(Arg280Met) | missense | no data | ex1 | yes | LPAT | PM2, PM5, PP3, PP4 | novel |
| EXT_ND_1 | ND | F | Sanger EXT1 | *EXT1* | chr8:119122447C>A | NM_000127.3:c.839G>T | NP_000118.2:p.(Arg280Met) | missense | no data | ex1 | yes | LPAT | PM2, PM5, PP3, PM1 | novel |
| EXT-45 | S | M | Sanger EXT1 | *EXT1* | chr8:119122448_119122449del | NM_000127.3:c.837_838del | NP_000118.2:p.(Arg280GlufsTer8) | frameshift | no data | ex1 |  | PAT | PM2, PVS1, PP4 | novel |
| EXT-105 | F | F | Sanger EXT1 | *EXT1* | chr8:119122451dup | NM_000127.3:c.835dup | NP_000118.2:p.(Thr279AsnfsTer10) | frameshift | no data | ex1 |  | PAT | PM2, PVS1, PP4 | novel |
| EXT-9 | S | M | Gene panel | *EXT1* | chr8:119122456G>T | NM_000127.3:c.830C>A | NP_000118.2:p.(Ser277Ter) | nonsense | no data | ex1 |  | PAT | PM2, PVS1, PP4, PP5 | CM099155 |
| EXT_ND_5 | ND | M | Gene panel | *EXT1* | chr8:119122466_119122467del | NM_000127.3:c.819_820del | NP_000118.2:p.(Gly274AspfsTer14) | frameshift | no data | ex1 |  | LPAT | PM2, PVS1 | novel |
| EXT-24 | ND | M | Gene panel | *EXT1* | chr8:119122474T>C | NM_000127.3:c.812A>G | NP_000118.2:p.(Tyr271Cys) | missense | no data | ex1 | yes | LPAT | PM2, PM5, PP3, PM1, PP5, PP5 | CM142837 |
| EXT-85 | F | M | Gene panel | *EXT1* | chr8:119122474T>C | NM_000127.3:c.812A>G | NP_000118.2:p.(Tyr271Cys) | missense | no data | ex1 | yes | LPAT | PM2, PM5, PP3, PM1, PP5, PP5 | CM142837 |
| EXT-70 | F | F | Sanger EXT1 | *EXT1* | chr8:119122474T>C | NM_000127.3:c.812A>G | NP_000118.2:p.(Tyr271Cys) | missense | no data | ex1 | yes | LPAT | PM2, PM5, PP3, PM1, PP5, PP5 | CM142837 |
| EXT-149 | S | F | Sanger EXT1 | *EXT1* | chr8:119122483C>A | NM_000127.3:c.803G>T | NP_000118.2:p.(Gly268Val) | missense | no data | ex1 |  | LPAT | PM2, PM5, PP3, PP4 | novel |
| EXT-220 | F | M | Gene panel | *EXT1* | chr8:119122484C>T | NM_000127.3:c.802G>A | NP_000118.2:p.(Gly268Arg) | missense | no data | ex1 | yes | LPAT | PM2, PM5, PP3, PM1, PP5, PP5 | CM189263 |
| EXT-199 | F | M | Gene panel | *EXT1* | chr8:119122484C>T | NM_000127.3:c.802G>A | NP_000118.2:p.(Gly268Arg) | missense | no data | ex1 | yes | LPAT | PM2, PM5, PP3, PM1, PP5, PP5 | CM189263 |
| EXT-136 | F | M | Sanger EXT1 | *EXT1* | chr8:119122484C>T | NM_000127.3:c.802G>A | NP_000118.2:p.(Gly268Arg) | missense | no data | ex1 | yes | LPAT | PM2, PM5, PP3, PM1, PP5, PP5 | CM189263 |
| EXT_ND_2 | ND | F | Sanger EXT1 | *EXT1* | chr8:119122515dup | NM_000127.3:c.771dup | NP_000118.2:p.(Pro258SerfsTer31) | frameshift | no data | ex1 |  | LPAT | PM2, PVS1 | novel |
| EXT-88 | S | M | Gene panel | *EXT1* | chr8:119122543del | NM_000127.3:c.747del | NP_000118.2:p.(Leu251Ter) | nonsense | no data | ex1 |  | PAT | PM2, PVS1, PP4, PP5 | novel |
| EXT-221 | F | M | Gene panel | *EXT1* | chr8:119122587dup | NM_000127.3:c.699dup | NP_000118.2:p.(Ile234TyrfsTer6) | frameshift | no data | ex1 |  | PAT | PM2, PVS1, PP4 | novel |
| EXT-46 | S | F | Sanger EXT1 | *EXT1* | chr8:119122620_119122642del | NM_000127.3:c.644_666del | NP_000118.2:p.(Met215AsnfsTer2) | frameshift | de novo | ex1 |  | PAT | PM2, PVS1, PS2, PP4 | novel |
| EXT-11 | F | F | Sanger EXT1 | *EXT1* | chr8:119122622_119122635delinsCAAA | NM_000127.3:c.651_664delinsTTTG | NP_000118.2:p.(Lys218LeufsTer31) | frameshift | no data | ex1 |  | PAT | PM2, PVS1, PP4 | novel |
| EXT-119 | S | F | Sanger EXT1 | *EXT1* | chr8:119122625_119122635del | NM_000127.3:c.651_661del | NP_000118.2:p.(Lys218GlnfsTer3) | frameshift | no data | ex1 |  | PAT | PM2, PVS1, PP4, PP5 | novel |
| EXT-222 | F | M | Gene panel | *EXT1* | chr8:119122649G>A | NM_000127.3:c.637C>T | NP_000118.2:p.(Gln213Ter) | nonsense | no data | ex1 | yes | PAT | PM2, PVS1, PP4, PP5 | CM1910282 |
| EXT-159 | F | M | Sanger EXT1 | *EXT1* | chr8:119122649G>A | NM_000127.3:c.637C>T | NP_000118.2:p.(Gln213Ter) | nonsense | maternal | ex1 | yes | PAT | PM2, PVS1, PP1, PP4, PP5 | CM1910282 |
| EXT-112 | F | F | Gene panel | *EXT1* | chr8:119122678T>G | NM_000127.3:c.608A>C | NP_000118.2:p.(Tyr203Ser) | missense | paternal | ex1 |  | LPAT | PM2, PM5, PP3, PM1, PP5, PP5 | CM2122567 |
| EXT-133 | S | F | Sanger EXT1 | *EXT1* | chr8:119122686C>T | NM_000127.3:c.600G>A | NP_000118.2:p.(Trp200Ter) | nonsense | maternal | ex1 | yes | PAT | PM2, PVS1, PP1, PP4, PP5 | CM980637 |
| EXT-146 | F | M | Sanger EXT1 | *EXT1* | chr8:119122686C>T | NM_000127.3:c.600G>A | NP_000118.2:p.(Trp200Ter) | nonsense | no data | ex1 | yes | PAT | PM2, PVS1, PP4, PP5 | CM980637 |
| EXT-223 | F | F | Gene panel | *EXT1* | chr8:119122693C>A | NM_000127.3:c.593G>T | NP_000118.2:p.(Gly198Val) | missense | no data | ex1 |  | VUS | PM2, PP3, PP4 | novel |
| EXT-72 | S | F | Sanger EXT1 | *EXT1* | chr8:119122751G>A | NM_000127.3:c.535C>T | NP_000118.2:p.(Gln179Ter) | nonsense | no data | ex1 | yes | PAT | PM2, PVS1, PP4, PP5 | CM065158 |
| EXT-139 | F | M | Gene panel | *EXT1* | chr8:119122751G>A | NM_000127.3:c.535C>T | NP_000118.2:p.(Gln179Ter) | nonsense | no data | ex1 | yes | PAT | PM2, PVS1, PP4, PP5 | CM065158 |
| EXT-171 | S | F | Sanger EXT1 | *EXT1* | chr8:119122793G>A | NM_000127.3:c.493C>T | NP_000118.2:p.(Gln165Ter) | nonsense | no data | ex1 | yes | PAT | PM2, PVS1, PP4, PP5 | CM053230 |
| EXT-224 | S | M | Gene panel | *EXT1* | chr8:119122793G>A | NM_000127.3:c.493C>T | NP_000118.2:p.(Gln165Ter) | nonsense | no data | ex1 | yes | PAT | PM2, PVS1, PP4, PP5 | CM053230 |
| EXT-12 | S | M | Sanger EXT1 | *EXT1* | chr8:119122826del | NM_000127.3:c.462del | NP_000118.2:p.(Phe154LeufsTer3) | frameshift | no data | ex1 |  | PAT | PM2, PVS1, PP4 | novel |
| EXT-104 | F | M | Gene panel | *EXT1* | chr8:119122837_119122838insAAGGCACGCCT | NM_000127.3:c.458_459insTAGGCGTGCCT | NP_000118.2:p.(Phe154ArgfsTer7) | frameshift | no data | ex1 |  | PAT | PM2, PVS1, PP4 | novel |
| EXT-48 | S | F | Gene panel | *EXT1* | chr8:119122866del | NM_000127.3:c.420del | NP_000118.2:p.(Ser141ProfsTer16) | frameshift | no data | ex1 |  | PAT | PM2, PVS1, PP4, PP5 | novel |
| EXT_ND_4 | ND | M | Gene panel | *EXT1* | chr8:119122882dup | NM_000127.3:c.404dup | NP_000118.2:p.(Ala136SerfsTer53) | frameshift | no data | ex1 |  | LPAT | PM2, PVS1 | novel |
| EXT-41 | F | F | Sanger EXT1 | *EXT1* | chr8:119122901C>A | NM_000127.3:c.385G>T | NP_000118.2:p.(Glu129Ter) | nonsense | no data | ex1 |  | PAT | PM2, PVS1, PP4, PP5 | CM189254 |
| EXT_ND_9 | ND | F | Sanger EXT1 | *EXT1* | chr8:119122916del | NM_000127.3:c.373del | NP_000118.2:p.(Glu125ArgfsTer11) | frameshift | no data | ex1 |  | LPAT | PM2, PVS1 | novel |
| EXT-32 | F | M | Gene panel | *EXT1* | chr8:119122922G>A | NM_000127.3:c.364C>T | NP_000118.2:p.(Gln122Ter) | nonsense | no data | ex1 | yes | PAT | PM2, PVS1, PP4, PP5 | CM065157 |
| EXT-191 | F | F | Sanger EXT1 | *EXT1* | chr8:119122922G>A | NM_000127.3:c.364C>T | NP_000118.2:p.(Gln122Ter) | nonsense | no data | ex1 | yes | PAT | PM2, PVS1, PP4, PP5 | CM065157 |
| EXT-174 | F | F | Sanger EXT1 | *EXT1* | chr8:119122939del | NM_000127.3:c.347del | NP_000118.2:p.(Val116AlafsTer20) | frameshift | no data | ex1 |  | PAT | PM2, PVS1, PP4 | novel |
| EXT-29 | S | F | Gene panel | *EXT1* | chr8:119122943T>A | NM_000127.3:c.343A>T | NP_000118.2:p.(Lys115Ter) | nonsense | no data | ex1 |  | PAT | PM2, PVS1, PP4 | novel |
| EXT-61 | S | M | Gene panel | *EXT1* | chr8:119122991_119122998del | NM_000127.3:c.289_296del | NP_000118.2:p.(Lys97HisfsTer89) | frameshift | no data | ex1 |  | PAT | PM2, PVS1, PP4 | novel |
| EXT-19 | F | M | Sanger EXT1 | *EXT1* | chr8:119123044dup | NM_000127.3:c.247dup | NP_000118.2:p.(Arg83ProfsTer106) | frameshift | no data | ex1 |  | PAT | PM2, PVS1, PP4, PP5 | CI972594 |
| EXT-134 | F | F | Gene panel | *EXT1* | chr8:119123056del | NM_000127.3:c.230del | NP_000118.2:p.(Ser77ThrfsTer59) | frameshift | no data | ex1 |  | PAT | PM2, PVS1, PP4 | novel |
| EXT-188 | F | M | Sanger EXT1 | *EXT1* | chr8:119123059del | NM_000127.3:c.228del | NP_000118.2:p.(Ser77AlafsTer59) | frameshift | no data | ex1 |  | PAT | PM2, PVS1, PP4 | novel |
| EXT-167 | S | M | Sanger EXT1 | *EXT1* | chr8:119123082C>T | NM_000127.3:c.204G>A | NP_000118.2:p.(Trp68Ter) | nonsense | no data | ex1 |  | PAT | PM2, PVS1, PP4, PP5 | CM980634 |
| EXT-116 | F | M | Sanger EXT1 | *EXT1* | chr8:119123087del | NM_000127.3:c.200del | NP_000118.2:p.(Pro67LeufsTer69) | frameshift | no data | ex1 |  | PAT | PM2, PVS1, PP4 | novel |
| EXT-160 | S | M | Gene panel | *EXT1* | chr8:119123091dup | NM_000127.3:c.195dup | NP_000118.2:p.(Val66ArgfsTer123) | frameshift | no data | ex1 |  | PAT | PM2, PVS1, PP4 | novel |
| EXT-201 | S | F | Gene panel | *EXT1* | chr8:119123102_119123111del | NM_000127.3:c.175_184del | NP_000118.2:p.(Pro59Cysfs\|74) | frameshift | no data | ex1 |  | PAT | PM2, PVS1, PP4 | novel |
| EXT-129 | S | M | Gene panel | *EXT1* | chr8:119123177_119123178del | NM_000127.3:c.112_113del | NP_000118.2:p.(Glu38ArgfsTer5) | frameshift | no data | ex1 |  | PAT | PM2, PVS1, PP4 | novel |
| EXT-62 | F | F | Sanger EXT1 | *EXT1* | chr8:119123204dup | NM_000127.3:c.84dup | NP_000118.2:p.(Arg29Ter) | nonsense | no data | ex1 |  | PAT | PM2, PVS1, PP4 | novel |
| EXT-89 | F | M | Sanger EXT1 | *EXT1* | chr8:119123240_119123241del | NM_000127.3:c.45_46del | NP_000118.2:p.(Cys16SerfsTer13) | frameshift | no data | ex1 |  | PAT | PM2, PVS1, PP4, PP5 | CD092515 |
| EXT-99 | F | M | Gene panel | *EXT1* | chr8:119123241dup | NM_000127.3:c.46dup | NP_000118.2:p.(Cys16LeufsTer14) | frameshift | no data | ex1 |  | PAT | PM2, PVS1, PP4 | novel |
| EXT-204 | S | M | Gene panel, MLPA | Negative |  |  |  |  |  |  |  |  |  |  |
| EXT-50 | S | M | Gene panel, MLPA | Negative |  |  |  |  |  |  |  |  |  |  |
| EXT-220 | S | M | Gene panel, MLPA | Negative |  |  |  |  |  |  |  |  |  |  |
| EXT-26 | S | F | Gene panel, MLPA | Negative |  |  |  |  |  |  |  |  |  |  |
| EXT-8 | S | F | Gene panel, MLPA | Negative |  |  |  |  |  |  |  |  |  |  |
| EXT-55 | S | M | Gene panel, MLPA | Negative |  |  |  |  |  |  |  |  |  |  |
| EXT-202 | S | F | Gene panel, MLPA | Negative |  |  |  |  |  |  |  |  |  |  |
| EXT-220 | S | F | Gene panel, MLPA | Negative |  |  |  |  |  |  |  |  |  |  |
| EXT-145 | S | F | Gene panel, MLPA | Negative |  |  |  |  |  |  |  |  |  |  |
| EXT-225 | S | F | Gene panel, MLPA | Negative |  |  |  |  |  |  |  |  |  |  |
| EXT-226 | S | M | Gene panel, MLPA | Negative |  |  |  |  |  |  |  |  |  |  |
| EXT-90 | S | M | Sanger EXT1/2, MLPA | Negative |  |  |  |  |  |  |  |  |  |  |
| EXT-148 | S | M | Gene panel, MLPA | Negative |  |  |  |  |  |  |  |  |  |  |
| EXT-150 | S | M | Gene panel, MLPA | Negative |  |  |  |  |  |  |  |  |  |  |
| EXT-15 | F | F | Sanger EXT1/2, MLPA | Negative |  |  |  |  |  |  |  |  |  |  |
| EXT-28 | S | M | Gene panel, MLPA | Negative |  |  |  |  |  |  |  |  |  |  |
| EXT-182 | S | M | Sanger EXT1/2, MLPA, gene panel | Negative |  |  |  |  |  |  |  |  |  |  |
| EXT-185 | S | M | Sanger EXT1/2, MLPA | Negative |  |  |  |  |  |  |  |  |  |  |
| EXT-31 | S | M | Gene panel, MLPA | Negative |  |  |  |  |  |  |  |  |  |  |
| EXT-110 | S | M | Gene panel, MLPA | Negative |  |  |  |  |  |  |  |  |  |  |
| EXT-168 | S | M | Sanger EXT1/2, MLPA, gene panel | Negative |  |  |  |  |  |  |  |  |  |  |
| EXT-82 | S | M | Sanger EXT1/2, MLPA | Negative |  |  |  |  |  |  |  |  |  |  |
| EXT-80 | S | F | Gene panel, MLPA | Negative |  |  |  |  |  |  |  |  |  |  |
| EXT-63 | S | M | Sanger EXT1/2, MLPA | Negative |  |  |  |  |  |  |  |  |  |  |

**Suppl. Table S2. Clinical and molecular data of 244 probands from unrelated families. Part B. Clinical Data**

| **Patient code #** | Gene | **Ancestry** | **Age of last examination** | **Age of discovery of 1st OC** | **Localisation of first discovered OC** | **IOR Scale 2013** | **IOR scale 2021** | **Additional findings** |
| --- | --- | --- | --- | --- | --- | --- | --- | --- |
| EXT-189 | *EXT2* | Russians | 3 | 0.5 | Ribs | IIA | IIA |  |
| EXT-16 | *EXT2* | Russians | 32 | 5 | Upper leg | IB | IB |  |
| EXT_ND_3 | *EXT2* | Russians | 32 | ND | ND | ND | ND | ND |
| EXT-83 | *EXT2* | Russians | 24 | 2 | Forearm | IIA | IIB |  |
| EXT-131 | *EXT2* | Russians | 6 | 0.3 | Multi | IB | IB |  |
| EXT-79 | *EXT2* | Russians | 24 | 3 | Forearm | IIIB | IIIB |  |
| EXT-155 | *EXT2* | Russians | 16 | ND | ND | IIIB | IIIB | Meningioma HP:0002858 (surgery at 12 y.o.), Focal-onset seizure HP:0007359 ( Following the surgery, the patient, previously experiencing multiple seizures, encountered only two post-operative episodes. Presently, the patient remains in remission.), Macrocephaly HP:0000256. |
| EXT-181 | *EXT2* | Russians | 15 | ND | Multi | IIA | IIB | Calf muscle hypertrophy HP:0008981, Hypermelanotic macule HP:0001034 on the abdomen. |
| EXT-73 | *EXT2* | Russians | 10 | 5 | Upper leg | IIA | IIA | Plexiform neurofibroma HP:0009732 of palm (brain MRI – without pathology, absent of Lisch nodules, absent of Cafe-au-lait spots) |
| EXT-20 | *EXT2* | Uzbeks | 20 | 2 | Arm | IIIB | IIIB |  |
| EXT-147 | *EXT2* | Kazakhs | 3 | 1 | Ribs | IB | IB |  |
| EXT-207 | *EXT2* | Russians | 7 | 1 | Scapula | IB | IB |  |
| EXT-5 | *EXT2* | Russians | 9 | 4 | Upper leg | IB | IB |  |
| EXT-165 | *EXT2* | Russians | 11 | 2 | Scapula | IIA | IIA |  |
| EXT-58 | *EXT2* | Russians | 7 | 1 | Forearm | IIA | IIB |  |
| EXT-2 | *EXT2* | Tatars | 6 | 5 | Multi | IB | IB | Hyperbilirubinemia (Gilbert syndrome): homozygous for 2 extra bases (TA) in the TATAA element of the 5-prime promoter region of the gene |
| EXT-38 | *EXT2* | Bashkirs | 11 | 3.5 | Arm | IB | IB |  |
| EXT-208 | *EXT2* | Russians | 5 | 1 | Scapula | IB | IB |  |
| EXT-67 | *EXT2* | Russians | 31 | ND | ND | IIA | IIA |  |
| EXT-140 | *EXT2* | Russians | 35 | 1.5 | Multi | IIA | IIB |  |
| EXT-18 | *EXT2* | Russians | 6 | 0.5 | Multi | IIA | IIA |  |
| EXT-10 | *EXT2* | Russians | 6 | 1 | Forearm | IIA | IIB |  |
| EXT-111 | *EXT2* | Russians | 4 | 1 | Ribs | IIA | IIA |  |
| EXT-166 | *EXT2* | Russians | 3 | 2 | Lower leg | IA | IA |  |
| EXT-120 | *EXT2* | Russians | 3 | 2 | Forearm | IB | IB | Hydronephrosis HP:0000126, right horseshoe kidney HP:0000085. Surgery at the age of 1 month |
| EXT-193 | *EXT2* | Russians | 8 | 7 | Knee | IB | IB |  |
| EXT-81 | *EXT2* | Russians | 58 | ND | ND | IIIA | IIIA |  |
| EXT-101 | *EXT2* | Russians | 14 | 0.6 | Multi | IIIB | IIIB |  |
| EXT-164 | *EXT2* | Russians | 5 | 1 | Ribs | IIA | IIB |  |
| EXT-7 | *EXT2* | Russians | 10 | ND | ND | IIA | IIB |  |
| EXT-93 | *EXT2* | Russians | 25 | 4 | Multi | IIA | IIB |  |
| EXT-65 | *EXT2* | Russians | 4 | 6 | Arm | IB | IB |  |
| EXT-206 | *EXT2* | Russians | 13 | 1 | Multi | IIIB | IIIB | High myopia HP:0011003, Astigmatism HP:0000483, Unilateral ptosis HP:0007687, Decreased nerve conduction velocity HP:0000762, Steppage gait HP:0003376, Pes cavus HP:0001761, Motor delay HP:0001270, Peripheral neuropathy HP:0009830. Hereditary motor and sensory neuropathy 1В caused by heterozygous missense substitution in the *MPZ* gene с.389A>G p.( Lysl30Arg) |
| EXT-22 | *EXT2* | Russians | 12 | 4 | Multi | IIA | IIA |  |
| EXT-173 | *EXT2* | Russians | 11 | 1 | Multi | IIA | IIA |  |
| EXT_ND_8 | *EXT2* | Belarusians | 31 | ND | ND | ND | ND | ND |
| EXT-122 | *EXT2* | Russians | 39 | 0.6 | Multi | IIB | IIB |  |
| EXT-68 | *EXT2* | Russians | 4 | 2 | Multi | IIA | IIB |  |
| EXT-97 | *EXT2* | Russians | 10 | 3.5 | Lower leg | IIA | IIB |  |
| EXT-128 | *EXT2* | Russians | 13 | 3 | Upper leg | IIA | IIB |  |
| EXT-194 | *EXT2* | Russians | 3 | 0.7 | Lower leg | IB | IB |  |
| EXT-53 | *EXT2* | Russians | 9 | 2 | Arm | IIA | IIB |  |
| EXT-1 | *EXT2* | Russians | 14 | 1 | Scapula | IIA | IIB | Congenital contracture of left hip HP:0002803 |
| EXT-162 | *EXT2* | Russians | 7 | 5 | Arm | IIA | IIA |  |
| EXT-115 | *EXT2* | Russians | 5 | 2.6 | Arm | IIA | IIB |  |
| EXT-54 | *EXT2* | Russians | 5 | 0.5 | Ribs | IB | IB |  |
| EXT-190 | *EXT2* | Armenians | 2 | 1 | Ribs | IA | IA |  |
| EXT-47 | *EXT2* | Armenians | 69 | ND | ND | IIIA | IIIA |  |
| EXT-84 | *EXT2* | Russians | 7 | ND | ND | IIA | IIA |  |
| EXT-64 | *EXT2* | Russians | 63 | 5 | Knee | IIIB | IIIB |  |
| EXT-209 | *EXT2* | Russians | 7 | 0.75 | Multi | IIA | IIA |  |
| EXT-195 | *EXT2* | Tatars | 6 | 0.75 | Multi | IIA | IIA | Iron deficiency anemia HP:0001891 |
| EXT-36 | *EXT2* | Ukrainians | 7 | 1.5 | Multi | IB | IB |  |
| EXT-49 | *EXT2* | Russians | 14 | 2 | Multi | IIA | IIB |  |
| EXT-172 | *PTPN11* | Russians | 6 | 2 | Lower leg | IB | IB |  |
| EXT-151 | *PTPN11* | Russians | 2 | 1.2 | Hands phalanges | IA | IA | Metopic synostosis HP:0011330, Epicanthus HP:0000286 |
| EXT-205 | *PTPN11* | Russians | 9 | 7 | Knee | IA | IA | DNA diagnosis was performed on skin biopsy material due to a bone marrow transplant at an early age. Primary immunodeficiency confirmed by molecular genetics, a hemizygous variant was detected by Sanger direct sequencing in the WAS gene, leading to a frameshift NM_000377.3:c.390delC p.(Asp130Glufs*131) Family history: first case in the family of bone neoplasms. First case in the family - primary immunodeficiency (Mother is a carrier of the variant in the WAS gene) |
| EXT-57 | *PTPN11* | Russians | 4 | 0 | Hands phalanges | IB | IB |  |
| EXT-13 | *PTPN11* | Tatars | 5 | 2.6 | Forearm | IIA | IIA |  |
| EXT-35 | *PTPN11* | Russians | 1 | 0.2 | Hands phalanges | IIA | IIA | Metopic synostosis HP:0011330, Iron deficiency anemia HP:0001891, Epicanthus HP:0000286 |
| EXT-184 | *PTPN11* | Russians | 2 | 1.5 | Lower leg | IIA | IIB | Attention deficit hyperactivity disorder HP:0007018 |
| EXT-170 | *PTPN11* | Russians | 9 | 0.6 | Hands phalanges | IIA | IIA |  |
| EXT-210 | *PTPN11* | Russians | 11 | 1.5 | Hands phalanges | IB | IB | Recurrent fractures HP:0002757 |
| EXT-75 | *EXT1* | Russians | 7 | 2 | Multi | IIA | IIA |  |
| EXT-211 | *EXT1* | Russians | 14 | ND | ND | IIA | IIB |  |
| EXT-117 | *EXT1* | Russians | 4 | 3 | Forearm | IIIB | IIIB | Delayed speech and language development HP:0000750, Macrocephaly HP:0000256. Additional de novo deletion 16p11.2: arr[hg19] 8q24.11(118660515_119031595)x1,16p11.2(29432212_30191848)x1 |
| EXT-87 | *EXT1* | Russians | 10 | 1.5 | Lower leg | IIIB | IIIB | Nail dystrophy HP:0008404 |
| EXT_ND_12 | *EXT1* | Russians | 6 | ND | ND | ND | ND | ND |
| EXT-107 | *EXT1* | Russians | 10 | 5 | Multi | IIA | IIA |  |
| EXT-212 | *EXT1* | Russians | 3 | 0.75 | Scapula | IB | IB | Subcutaneous lipoma HP:0001031, Hepatomegaly HP:0002240, Recurrent pyelonephritis HP:0012787 |
| EXT-183 | *EXT1* | Russians | 27 | ND | ND | IIIB | IIIB | Elbow congenital contracture HP:0002803, enchondroma of hand phalanges HP:0005701 |
| EXT-186 | *EXT1* | Russians | 3 | ND | Forearm | IIA | IIA |  |
| EXT-95 | *EXT1* | Russians | 7 | 2 | Forearm | IB | IB |  |
| EXT-69 | *EXT1* | Russians | 5 | ND | ND | IB | IB |  |
| EXT-30 | *EXT1* | Russians | 13 | 1.5 | Multi | IIIB | IIIB |  |
| EXT-103 | *EXT1* | Ukrainians | 30 | 21 | Knee | IA | IA |  |
| EXT-77 | *EXT1* | Russians | 15 | 15 | Ribs | IB | IB | Osteosarcoma HP:0002669 of rib at 15 years old |
| EXT-114 | *EXT1* | Azerbaijani | 3 | 1.5 | Scapula | IB | IB | Unconjugated hyperbilirubinemia HP:0008282 (Gilbert syndrome), homozygous for 2 extra bases (TA) in the TATAA element of the 5-prime promoter region of the gene |
| EXT-126 | *EXT1* | Russians | 28 | 13 | Forearm | IIB | IIB |  |
| EXT_ND_13 | *EXT1* | Uzbeks | 6 | ND | ND | ND | ND | ND |
| EXT-92 | *EXT1* | Russians | 31 | ND | Arm | IB | IB | Unconjugated hyperbilirubinemia HP:0008282 (Gilbert syndrome), homozygous for 2 extra bases (TA) in the TATAA element of the 5-prime promoter region of the gene |
| EXT-23 | *EXT1* | Jews | 3 | 2.75 | Knee | IA | IA |  |
| EXT-52 | *EXT1* | Russians | 16 | 7 | Knee | IIA | IIB |  |
| EXT-56 | *EXT1* | Russians | 4 | 4 | Multi | IB | IB | Scapular winging HP:0003691 without any osteochondroma in scapular area |
| EXT-192 | *EXT1* | Russians | 33 | ND | ND | IIA | IIB |  |
| EXT_ND_7 | *EXT1* | Russians | 4 | ND | ND | ND | ND | ND |
| EXT-25 | *EXT1* | Russians | 6 | 0.5 | Ribs | IIA | IIB |  |
| EXT-137 | *EXT1* | Russians | 5 | 4.5 | Forearm | IB | IB |  |
| EXT-96 | *EXT1* | Russians | 4 | 2 | Ribs | IB | IB |  |
| EXT-141 | *EXT1* | Tatars | 3 | 0.6 | Forearm | IA | IA | Thrombocytopenia HP:0001873, Hemangiomatosis HP:0007461 |
| EXT-109 | *EXT1* | Russians | 4 | 3 | Multi | IB | IB |  |
| EXT-177 | *EXT1* | Ukrainians | 3 | ND | ND | IIA | IIB |  |
| EXT-21 | *EXT1* | Tatars | 5 | 5 | Arm | IA | IA |  |
| EXT-4 | *EXT1* | Russians | 1 | 0.8 | Ribs | IA | IA |  |
| EXT-76 | *EXT1* | Russians | 17 | 1 | Upper leg | IIA | IIB |  |
| EXT-130 | *EXT1* | Russians | 5 | ND | ND | IB | IB |  |
| EXT-51 | *EXT1* | Russians | 6 | 2 | Knee | IIB | IIB |  |
| EXT-124 | *EXT1* | Russians | 14 | 4 | Lower leg | IA | IA | Delayed speech and language development HP:0000750 |
| EXT-203 | *EXT1* | Russians | 6 | 0.5 | Arm | IIA | IIB |  |
| EXT_ND_14 | *EXT1* | Russians | 5 | ND | ND | ND | ND | ND |
| EXT-213 | *EXT1* | Arminian | 20 | 1 | Multi | IIIB | IIIB |  |
| EXT-158 | *EXT1* | Russians | 36 | 0.5 | Ribs | IIA | IIB |  |
| EXT-123 | *EXT1* | Bashkirs | 10 | 1 | Ribs | IB | IB |  |
| EXT-91 | *EXT1* | Russians | 7 | 3 | Forearm | IB | IB |  |
| EXT-113 | *EXT1* | Russians | 38 | 3 | Upper leg | IIA | IIB |  |
| EXT-27 | *EXT1* | Russians | 10 | 0.7 | Ribs | IB | IB |  |
| EXT-157 | *EXT1* | Russians | 17 | 3 | Hands phalanges | IIA | IIB |  |
| EXT-179 | *EXT1* | Russians | 11 | 0.25 | Hands phalanges | IIIA | IIIA |  |
| EXT-42 | *EXT1* | Russians | 9 | 1 | Ribs | IIA | IIB |  |
| EXT-100 | *EXT1* | Italians | 8 | 2 | Multi | IB | IB |  |
| EXT-215 | *EXT1* | Russians | 12 | 8 | Ribs | IB | IB |  |
| EXT-214 | *EXT1* | Russians | 8 | 2 | Forearm | IIA | IIB |  |
| EXT-216 | *EXT1* | Russians | 11 | 1 | Arm | IB | IB |  |
| EXT-71 | *EXT1* | Russians | 10 | 1 | Ribs | IB | IB |  |
| EXT-40 | *EXT1* | Russians | 13 | 1.25 | Ribs | IIA | IIB |  |
| EXT-43 | *EXT1* | Russians | 17 | 0.25 | Ribs | IIA | IIB | Horizontal nystagmus HP:0000666, Nail dystrophy HP:0008404 |
| EXT-132 | *EXT1* | Russians | 8 | 3 | Arm | IIA | IIA |  |
| EXT-175 | *EXT1* | Russians | 13 | 1.5 | Hands phalanges | IIA | IIB |  |
| EXT-180 | *EXT1* | Russians | 8 | 0 | Hands phalanges | IIA | IIA | Epistaxis HP:0000421, Thrombocytopenia HP:0001873 |
| EXT_ND_15 | *EXT1* | Russians | 2 | ND | ND | ND | ND | ND |
| EXT-217 | *EXT1* | Russians | 10 | 0.25 | Ribs | IB | IB |  |
| EXT-156 | *EXT1* | Russians | 9 | 1 | Ribs | IIA | IIB |  |
| EXT-169 | *EXT1* | Uzbeks | 29 | 3 | Ribs | IIIB | IIIB |  |
| EXT-102 | *EXT1* | Russians | 11 | 5 | Multi | IIA | IIB | Obesity HP:0001513, Type II diabetes mellitus HP:0005978 |
| EXT-118 | *EXT1* | Russians | 16 | 1.8 | Multi | IIA | IIB |  |
| EXT-135 | *EXT1* | Russians | 14 | 0 | Ribs | IIIA | IIIA |  |
| EXT-125 | *EXT1* | Russians | 9 | 1 | Ribs | IB | IB |  |
| EXT-176 | *EXT1* | Russians | 37 | 5 | Knee | IIA | IIA |  |
| EXT-66 | *EXT1* | Russians | 9 | 1 | Upper leg | IB | IB |  |
| EXT_ND_11 | *EXT1* | Russians | 8 | ND | ND | ND | ND | ND |
| EXT-17 | *EXT1* | Russians | 10 | 1 | Scapula | IB | IB |  |
| EXT_ND_16 | *EXT1* | Russians | 34 | ND | ND | ND | ND | ND |
| EXT-14 | *EXT1* | Russians | 6 | 2 | Knee | IB | IB | Myoclonic seizure HP:0032794 onset at 3-month-old, positive response on anticonvulsive therapy. Difficulty walking HP:0002355. Brain MRI - cyst HP:0030724 of the parietal region |
| EXT-178 | *EXT1* | Russians | 27 | ND | ND | IIIB | IIIB |  |
| EXT-74 | *EXT1* | Russians | 5 | 5 | Knee | IB | IB |  |
| EXT-138 | *EXT1* | Russians | 11 | 1 | Arm | IIB | IIB |  |
| EXT-108 | *EXT1* | Russians | 5 | ND | ND | IB | IB | Sparse hair HP:0008070, Cognitive impairment HP:0100543, Micrognathia HP:0000347, Thin upper lip vermilion HP:0000219 |
| EXT-33 | *EXT1* | Russians | 15 | 1 | Knee | IIA | IIA |  |
| EXT-196 | *EXT1* | Russians | 13 | 0.5 | Forearm | IB | IB |  |
| EXT-161 | *EXT1* | Russians | 24 | 0.7 | Ribs | IIA | IIA |  |
| EXT-39 | *EXT1* | Russians | 10 | 6 | Arm | IIA | IIA |  |
| EXT-153 | *EXT1* | Russians | 8 | 2 | Arm | IIA | IIA |  |
| EXT-3 | *EXT1* | Russians | 7 | 2 | Scapula | IB | IB |  |
| EXT-142 | *EXT1* | Russians | 35 | ND | ND | IIA | IIB |  |
| EXT-218 | *EXT1* | Russians | 10 | 3 | Hands phalanges | IIA | IIA |  |
| EXT-163 | *EXT1* | Russians | 11 | 5 | Knee | IIA | IIB | Hyperthyroidism HP:0000836, Obesity HP:0001513, |
| EXT_ND_6 | *EXT1* | Russians | 22 | ND | ND | ND | ND | ND |
| EXT_ND_17 | *EXT1* | Chuvash | 44 | ND | ND | ND | ND | ND |
| EXT-60 | *EXT1* | Russians | 29 | 2 | Arm | IIIA | IIIA |  |
| EXT-154 | *EXT1* | Russians | 5 | 0.5 | Multi | IB | IB | Cognitive impairment HP:0100543 |
| EXT-187 | *EXT1* | Russians | 31 | ND | ND | IIA | IIA |  |
| EXT-121 | *EXT1* | Russians | 12 | 0.5 | Lower leg | IIA | IIB |  |
| EXT-143 | *EXT1* | Karelians | 6 | 3 | Lower leg | IIA | IIB |  |
| EXT-59 | *EXT1* | Russians | 15 | 0.6 | Forearm | IIIB | IIIB |  |
| EXT-98 | *EXT1* | Russians | 28 | 12 | Knee | IIIA | IIIA |  |
| EXT-6 | *EXT1* | Russians | 40 | 1.5 | Multi | IIA | IIA |  |
| EXT-127 | *EXT1* | Russians | 8 | 0.25 | Forearm | IB | IB |  |
| EXT-219 | *EXT1* | Russians | 7 | 5 | Multi | IB | IB |  |
| EXT-94 | *EXT1* | Russians | 6 | ND | Knee | IIA | IIA |  |
| EXT-106 | *EXT1* | Russians | 33 | ND | ND | IIIB | IIIB |  |
| EXT_ND_18 | *EXT1* | Russians | 15 | ND | ND | ND | ND | ND |
| EXT-197 | *EXT1* | Chuvash | 3 | 7 | Ribs | IB | IB |  |
| EXT-44 | *EXT1* | Russians | 14 | 4 | Forearm | IIA | IIA |  |
| EXT-37 | *EXT1* | Russians | 10 | ND | ND | IB | IB | Focal-onset seizure HP:0007359 and Hearing impairment HP:0000365 after head injury in 8 year. Positive response on anticonvulsive therapy |
| EXT-78 | *EXT1* | Russians | 7 | 4.5 | Multi | IIIA | IIIA |  |
| EXT-152 | *EXT1* | Russians | 26 | 5 | Lower leg | IIIA | IIIA |  |
| EXT_ND_10 | *EXT1* | Russians | 32 | ND | ND | ND | ND | ND |
| EXT-34 | *EXT1* | Russians | 4 | 1 | Scapula | IB | IB |  |
| EXT-198 | *EXT1* | Russians | 8 | 5 | Forearm | IIA | IIB |  |
| EXT-144 | *EXT1* | Russians | 30 | 4 | Multi | IB | IB |  |
| EXT_ND_1 | *EXT1* | Russians | 5 | ND | ND | ND | ND | ND |
| EXT-45 | *EXT1* | Russians | 5 | 2 | Forearm | IB | IB |  |
| EXT-105 | *EXT1* | Russians | 36 | 5 | Multi | IB | IB |  |
| EXT-9 | *EXT1* | Russians | 2 | 3 | Arm | IA | IA |  |
| EXT_ND_5 | *EXT1* | Jews | 36 | ND | ND | ND | ND | ND |
| EXT-24 | *EXT1* | Russians | 17 | 5 | Knee | IB | IB |  |
| EXT-85 | *EXT1* | Russians | 7 | 2 | Ribs | IIA | IIA |  |
| EXT-70 | *EXT1* | Russians | 28 | ND | ND | IIA | IIB |  |
| EXT-149 | *EXT1* | Russians | 29 | 3 | Forearm | IIA | IIB |  |
| EXT-220 | *EXT1* | Russians | 4 | 3 | Multi | IB | IB |  |
| EXT-199 | *EXT1* | Armenians | 12 | 2 | Knee | IIA | IIA |  |
| EXT-136 | *EXT1* | Russians | 11 | 1 | Forearm | IIIA | IIIA |  |
| EXT_ND_2 | *EXT1* | Russians | 12 | ND | ND | ND | ND | ND |
| EXT-88 | *EXT1* | Russians | 1 | 0.5 | Ribs | IA | IA |  |
| EXT-221 | *EXT1* | Russians | 21 | 0.5 | Arm | IIIA | IIIA |  |
| EXT-46 | *EXT1* | Russians | 2 | 0.75 | Upper leg | IB | IB | Slow-growing hair HP:0002217, Split nail HP:0001809 |
| EXT-11 | *EXT1* | Tatars | 15 | 7 | Clavicula | IIA | IIA |  |
| EXT-119 | *EXT1* | Russians | 12 | 2 | Forearm | IB | IB |  |
| EXT-222 | *EXT1* | Russians | 9 | 0 | Arm | IIA | IIB |  |
| EXT-159 | *EXT1* | Russians | 17 | 2 | Scapula | IIIB | IIIB | Chronic fatigue HP:0012432, Attention deficit hyperactivity disorder HP:0007018 |
| EXT-112 | *EXT1* | Karachays | 6 | 0.5 | Scapula | IB | IB |  |
| EXT-133 | *EXT1* | Russians | 11 | 1 | Upper leg | IB | IB |  |
| EXT-146 | *EXT1* | Russians | 5 | ND | ND | IIIA | IIIA |  |
| EXT-223 | *EXT1* | Tatars | 9 | ND | ND | IIA | IIA |  |
| EXT-72 | *EXT1* | Russians | 35 | 2 | Ribs | IB | IB |  |
| EXT-139 | *EXT1* | Kazakhs | 9 | 7 | Multi | IIA | IIB |  |
| EXT-171 | *EXT1* | Russians | 28 | 3 | Multi | IIA | IIA |  |
| EXT-224 | *EXT1* | Russians | 8 | 2 | Knee | IIA | IIB |  |
| EXT-12 | *EXT1* | Russians | 11 | 0.25 | Forearm | IIIB | IIIB |  |
| EXT-104 | *EXT1* | Russians | 4 | 1 | Knee | IB | IB |  |
| EXT-48 | *EXT1* | Russians | 1 | 0.75 | Ribs | IA | IA | Enchondroma HP:0030038 |
| EXT_ND_4 | *EXT1* | Lezgin | 16 | ND | ND | ND | ND | ND |
| EXT-41 | *EXT1* | Russians | 14 | 3 | Multi | IIB | IIB |  |
| EXT_ND_9 | *EXT1* | Avars | 3 | ND | ND | ND | ND | ND |
| EXT-32 | *EXT1* | Russians | 17 | 4 | Forearm | IB | IB |  |
| EXT-191 | *EXT1* | Russians | 23 | ND | ND | IIA | IIA |  |
| EXT-174 | *EXT1* | Russians | 38 | 2.5 | Multi | IIA | IIB |  |
| EXT-29 | *EXT1* | Russians | 8 | 6 | Forearm | IB | IB |  |
| EXT-61 | *EXT1* | Uzbeks | 13 | 10 | Knee | IIIA | IIIA |  |
| EXT-19 | *EXT1* | Russians | 51 | 3 | Upper leg | IIA | IIA |  |
| EXT-134 | *EXT1* | Russians | 40 | 2.5 | Ribs | IIA | IIA |  |
| EXT-188 | *EXT1* | Belarusians | 16 | 1.5 | Lower leg | IIA | IIB |  |
| EXT-167 | *EXT1* | Russians | 2 | 1 | Knee | IB | IB |  |
| EXT-116 | *EXT1* | Russians | 34 | ND | ND | IB | IB |  |
| EXT-160 | *EXT1* | Russians | 10 | 2 | Knee | IIA | IIA |  |
| EXT-201 | *EXT1* | White South Africans | 4 | 0.75 | Scapula | IIA | IIA |  |
| EXT-129 | *EXT1* | Russians | 4 | ND | ND | IB | IB | Delayed speech and language development HP:0000750 after infection, Episodic abdominal pain HP:0002574 |
| EXT-62 | *EXT1* | Russians | 34 | ND | ND | IB | IB |  |
| EXT-89 | *EXT1* | Russians | 9 | 2 | Multi | IIIA | IIIA |  |
| EXT-99 | *EXT1* | Russians | 12 | 1.5 | Multi | IIIA | IIIA |  |
| EXT-204 | Negative | Russians | 5 | 3.5 | Forearm | IA | IA |  |
| EXT-50 | Negative | Tatars | 7 | 2 | Forearm | IA | IA |  |
| EXT-220 | Negative | Russians | 19 | 8 | Knee | IA | IA |  |
| EXT-26 | Negative | Russians | 8 | 7 | Knee | IA | IA |  |
| EXT-8 | Negative | Russians | 15 | 0.75 | Lower leg | IA | IA | Lumbar hypertrichosis HP:0011913 |
| EXT-55 | Negative | Russians | 10 | 8 | Multi | IA | IA |  |
| EXT-202 | Negative | Russians | 2 | ND | ND | IA | IA |  |
| EXT-220 | Negative | Tatars | 13 | 10 | Upper leg | IA | IA |  |
| EXT-145 | Negative | Laks | 7 | ND | Arm | IB | IB |  |
| EXT-225 | Negative | Tatars | 5 | 3 | Arm | IB | IB |  |
| EXT-226 | Negative | Russians | 8 | 4 | Arm | IB | IB |  |
| EXT-90 | Negative | Kyrgyz | 4 | 2 | Forearm | IB | IB |  |
| EXT-148 | Negative | Russians | 6 | 4 | Hands phalanges | IB | IB |  |
| EXT-150 | Negative | Russians | 6 | 4 | Upper leg | IB | IB |  |
| EXT-15 | Negative | Russians | 25 | 4 | Forearm | IIA | IIA |  |
| EXT-28 | Negative | Russians | 2 | 1 | Multi | IIA | IIA |  |
| EXT-182 | Negative | Russians | 2 | ND | Ribs | IIA | IIA |  |
| EXT-185 | Negative | Jews | 10 | ND | Ribs | IIA | IIA |  |
| EXT-31 | Negative | Russians | 8 | 3 | Ribs | IIA | IIA |  |
| EXT-110 | Negative | Russians | 2 | 1.5 | Scapula | IIA | IIA | Recurrent fractures HP:0002757 |
| EXT-168 | Negative | Uzbeks | 6 | 3 | Upper leg | IIA | IIA |  |
| EXT-82 | Negative | Russians | 11 | 3 | Knee | IIA | IIB |  |
| EXT-80 | Negative | Chuvash | 29 | 4 | Pelvis | IIA | IIB |  |
| EXT-63 | Negative | Russians | 44 | 4 | Multi | IIIB | IIIB |  |
