# Supplementary material for: The Missing Piece of the Puzzle: Unveiling the Role of PTPN11 Gene in Multiple Osteochondromas in a Large Cohort Study
Source: Hum Mutat. 2024 Feb 12;2024:8849348. doi: 10.1155/2024/8849348 (PMC11918999; doi:10.1155/2024/8849348)
Supplement: Supplementary 3 — Table S3: clinical and molecular data from persons without any sights of osteochondromas from our local database with loss-of-function variant in the EXT2 gene. [file 8849348.f3.docx]

**Suppl. Table S3. Clinical and molecular data from persons without any sights of osteochondromas from our local database with loss-of-function variant in the EXT2 gene. Part A**

| **Individuals code** | **Genetic test** | **Diagnose** | **Additional physical examination for MO** | **Gender** | **Age** | **Gene** | **HGVS_Genomic_GRCh37** | **HGVS_transcript** | **HGVS_Predicted_Protein** | **Exon #** | **Type** | **Allele frequency in local data set** | | **Allele Count (gnomAD v2.1.1)** | **HGMD v.2022.1 ID #** | **Comments on SNV** | **SNV in the *EXT2* gene explain the initial diagnosis?** | **Does SNV in *EXT2* cause MO?** | **Comments on clinical data related to MO** |
| --- | --- | --- | --- | --- | --- | --- | --- | --- | --- | --- | --- | --- | --- | --- | --- | --- | --- | --- | --- |
| NonEXT-1 | Clinical exome sequencing | Oculocutaneous albinism | No response was received via phone and email | F | 4 | *EXT2* | chr11:44129329C>T | NM_207122.1:c.67C>T | p.Arg23Ter | ex2 | Nonsense | 1 / 51 214 | 1.95E-05 | 3 | CM980644 | The p.Arg23* found in many MO cohort studies before, also found in one proband from our cohort. This SNV is pathogenic. It is worth to mention it is one of the most known frequent pathogenic SNV in gnomAD data set among EXT1/2 genes | no | Too young for evaluation | Current status for MO is unknown |
| NonEXT-2 | Clinical exome sequencing | Multiple fetal anomalies, Meckel Gruber syndrome symptoms? | No response was received via phone and email | F | Fetus 13-14 week of pregnancy | *EXT2* | chr11:44129474del | NM_207122.1:c.212del | p.Leu71fs | ex2 | Fraimshift | 1 / 51 214 | 1.95E-05 | NA | novel | The p.Leu71fs is likely pathogenic variant and did not differ from other LoF variants in our and other MO cohorts before exon 9, also it was single submitted to Clinvar database as cause for MO | no | Too young for evaluation | Current status for MO is unknown. Also unknown if this fetus was born. |
| NonEXT-3 | Whole exome sequencing | Metabolic encephalopathy | Refused | F | 3 | *EXT2* | chr11:44219466C>T | NM_207122.1:c.1393C>T | p.Arg465Ter | ex9 | Nonsense | 1 / 51 214 | 1.95E-05 | 3 | CM1827419 | The p.Arg465* was found in patient with bladder urothelial carcinoma and in another study about results of clinical exome sequencing. In both papers status for MO for both cases didn't discussed, and all data located only in supplementary files. | no | Too young for evaluation | Current status for MO is unknown. From the phone call, the family didn't find anything that could be MO-related. The end response was: if the family finds any bone abnormality they will reach us. |
| NonEXT-4 | Clinical exome sequencing | Carrier screening | Refused | F | 33 | *EXT2* | chr11:44228373T>G | NM_207122.1:c.1526T>G | p.Leu509Ter | ex10 | Nonsense | 1 / 51 214 | 1.95E-05 | 1 | novel |  | No known mendelian disease | Unknown | Current status for MO is unknown. We didn’t have any medical records about the person |
| NonEXT-5 | Clinical exome sequencing | Healthy mother underwent CES as part of a trio analysis for her fetus with fetal polycystic kidney disease, anhydramnios | No response was received via phone and email | F | 38 | *EXT2* | chr11:44257852C>T | NM_207122.1:c.1945C>T | p.Arg649Ter | ex13 | Nonsense | 2 / 51 214 | 3.91E-05 | 9 | CM092514 | The p.Arg649* was found as the cause of MO only in one paper Heinritz et al. 2009. He has higher frequency in gnomAD as could be expected for MO. Also was found twice in our dataset. Allele with SNV predicted to escape NMD | No known mendelian disease | Unknown | Medical records about pregnancy didn't mention any OCs or relative to MO findings |
| NonEXT-6 | Whole exome sequencing | Healthy mother underwent WES as part of a trio analysis for her child with epilepsy | Yes, without OCs findings | F | 35 | *EXT2* | chr11:44257852C>T | NM_207122.1:c.1945C>T | p.Arg649Ter | ex13 | Nonsense | 2 / 51 214 | 3.91E-05 | 9 | CM092514 | The p.Arg649* was found as the cause of MO only in one paper Heinritz et al. 2009. He has higher frequency in gnomAD as could be expected for MO. Also was found twice in our dataset. Allele with SNV predicted to escape NMD | No known mendelian disease | No | Person didn't have any signs of MO or relative to MO findings and family history. The son of individual #NonEXT-6  with epilepsy  did not inherit the SNV from her and also did not exhibit any clinical features of "Seizures, scoliosis, and macrocephaly syndrome (OMIM#616682)", which is caused by biallelic variants in the EXT2 gene. |
| NonEXT-7 | Clinical exome sequencing | Torsion dystonia | Yes, without OCs findings | M | 17 | *EXT2* | chr11:44257926G>T | NM_207122.1:c.2018+1G>T | p.? | in13 | Splicing | 1 / 51 214 | 1.95E-05 | NA | novel | Allele with SNV predicted to escape NMD | No | No | Person didn't have any signs of MO or relative to MO findings and family history. |
| NonEXT-8 | Whole exome sequencing | Neurodegenerative disease? mild galactosemia | Refused | M | 19 | *EXT2* | chr11:44265763C>T | NM_207122.1:c.2083C>T | p.Arg728Ter | ex14 | Nonsense | 1 / 51 214 | 1.95E-05 | 3 | novel | Allele with SNV predicted to escape NMD | No | Unknown | Medical record has not mentioned any OCs  or relative to MO findings. By phone we also received vocal information that no one in person family had MO |

**Suppl. Table S3. Clinical and molecular data from persons without any sights of osteochondromas from our local database with loss-of-function variant in the EXT2 gene. Part B. Findings in different genes related to initial diagnose.**

| **Gender** | **Age** | **Gene** | **HGVS_Genomic_GRCh37** | **HGVS_transcript** | **HGVS_Predicted_Protein** | **Type** | **Exon** | **Allele Count (gnomAD)** | **ACMG critera** | **ACMG class** | **HGMD v.2022.1 ID #** |
| --- | --- | --- | --- | --- | --- | --- | --- | --- | --- | --- | --- |
| F | Fetus 13-14 week of pregnancy | *TMEM67* | chr8:94798481G>A | NM_153704.6:c.1319G>A | NP_714915.3:p.(Arg440Gln) | missense | 13 | 17 | PM2, PP3, PP5 | VUS | CM072086 |
|  |  | *TMEM67* | chr8:94792932dup | NM_153704.6:c.826dup | NP_714915.3:p.(Glu276GlyfsTer47) | frameshift | 8 | NA | PM2, PVS1 | LPAT | novel |
| M | 17 | *THAP1* | chr8:42694498C>T | NM_018105.3:c.98G>A | NP_060575.1:p.(Cys33Tyr) | missense | 2 | NA | PM2, PM5, PP3 | VUS | novel |
| M | 19 | *GALT* | chr9:34648167A>G | NM_000155.4:c.563A>G | NP_000146.2:p.(Gln188Arg) | missense | 6 | 412 | PM2, PP3, PS3, PP5 | LPAT | CM910169 |
|  |  | *GALT* | chr9:34649029G>T | NM_000155.4:c.855G>T | NP_000146.2:p.(Lys285Asn) | missense | 9 | 36 | PM2, PM5, PP3, PP5 | LPAT | CM920296 |
| F | 4 | negative findings | | | | | | | | | |
| F | 3 | negative findings | | | | | | | | | |
| F | 33 | several SNVs for AR diseases in the status of a healthy carrier | | | | | | | | | |
| F | 38 | several SNVs for AR diseases in the status of a healthy carrier | | | | | | | | | |
| F | 35 | several SNVs for AR diseases in the status of a healthy carrier | | | | | | | | | |
